# Relative importance of population size, fishing pressure and temperature on the spatial distribution of nine Northwest Atlantic groundfish stocks

**DOI:** 10.1371/journal.pone.0196583

**Published:** 2018-04-26

**Authors:** Charles F. Adams, Larry A. Alade, Christopher M. Legault, Loretta O’Brien, Michael C. Palmer, Katherine A. Sosebee, Michele L. Traver

**Affiliations:** National Marine Fisheries Service, Northeast Fisheries Science Center, Woods Hole, Massachusetts, United States of America; Havforskningsinstituttet, NORWAY

## Abstract

The spatial distribution of nine Northwest Atlantic groundfish stocks was documented using spatial indicators based on Northeast Fisheries Science Center spring and fall bottom trawl survey data, 1963–2016. We then evaluated the relative importance of population size, fishing pressure and bottom temperature on spatial distribution with an information theoretic approach. Northward movement in the spring was generally consistent with prior analyses, whereas changes in depth distribution and area occupancy were not. Only two stocks exhibited the same changes in spatiotemporal distribution in the fall as compared with the spring. Fishing pressure was the most important predictor of the center of gravity (i.e., bivariate mean location of the population) for the majority of stocks in the spring, whereas in the fall this was restricted to the east-west component. Fishing pressure was also the most important predictor of the dispersion around the center of gravity in both spring and fall. In contrast, biomass was the most important predictor of area occupancy for the majority of stocks in both seasons. The relative importance of bottom temperature was ranked highest in the fewest number of cases. This study shows that fishing pressure, in addition to the previously established role of climate, influences the spatial distribution of groundfish in the Northwest Atlantic. More broadly, this study is one of a small but growing body of literature to demonstrate that fishing pressure has an effect on the spatial distribution of marine resources. Future work must consider both fishing pressure and climate when examining mechanisms underlying fish distribution shifts.

## Introduction

Center of gravity (CG) metrics have been used in fisheries to examine density-dependent and temperature effects on the mean location of fish populations for several decades (e.g. [1–2]). More recently, time-series analyses of CG indices have documented latitudinal and depth shifts in response to warming for numerous species in the North Sea [3–5], the Northwest Atlantic [6] and the Bering Sea [7]. Changes in the CG of marine species assemblages have also been linked to climate velocities in nine regions of the continental shelves of North America [8–9].

The effect of fishing pressure on the CG has also been examined. Two of the aforementioned studies found no link between stock assessment model estimates of fishing mortality and changes in the CG [4–5]. Using an exploitation index (landings/survey biomass), Nye et al. [10] were unable to establish a clear link between fishing pressure and the CG of silver hake (*Merluccius bilinearis*) in the Northwest Atlantic. In contrast, other analyses have found that fishing mortality, in addition to climate, has an effect on the CG and depth indices for North Sea sole, *Solea solea* [11], whereas for North Sea cod (*Gadus morhua*), fishing mortality has an effect on only the east-west component of the CG [12].

**Fig 1:**
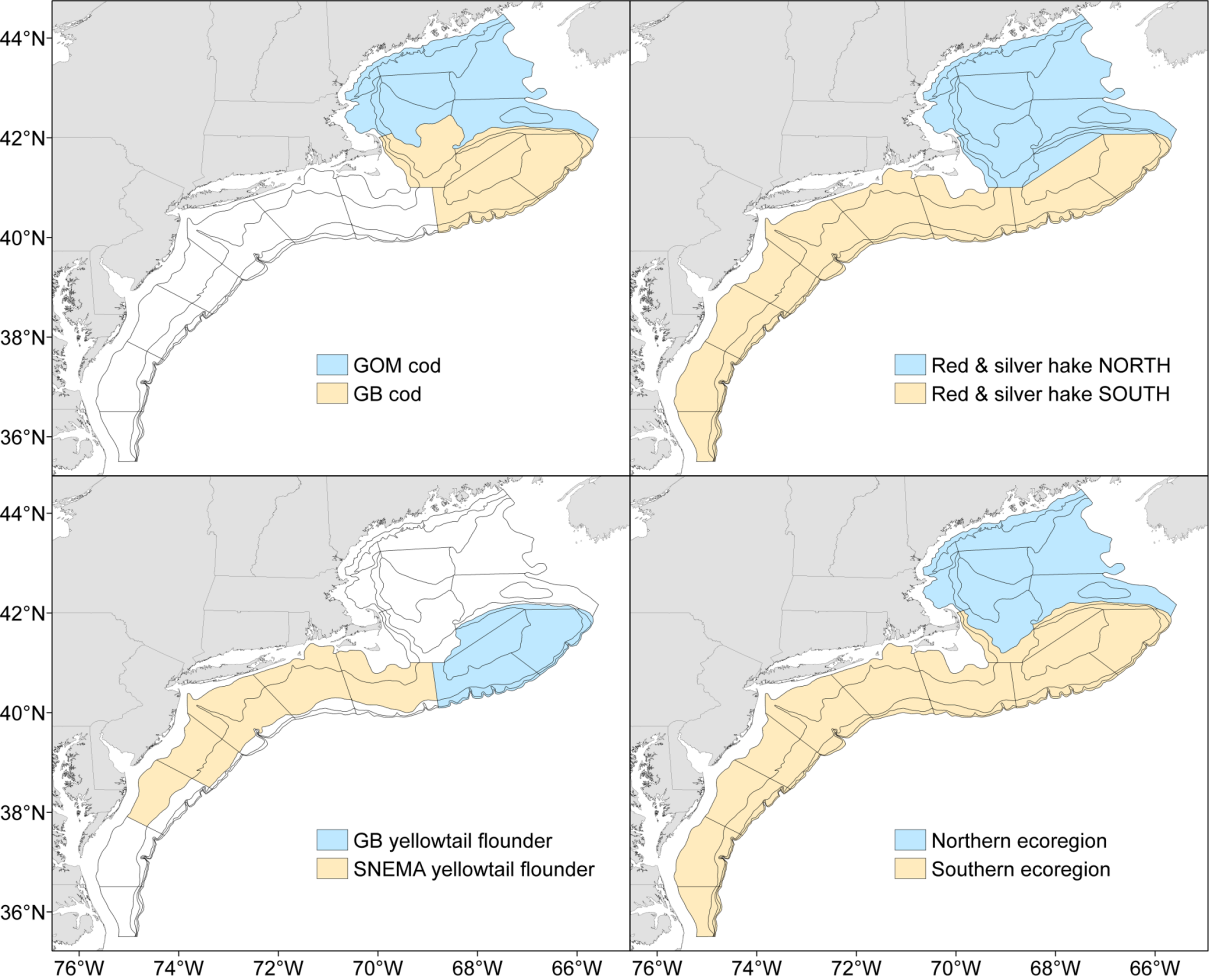
Northeast Fisheries Science Center bottom trawl survey offshore strata. Stock strata definitions are shown for Gulf of Maine and Georges Bank cod (upper left), the northern and southern stocks of red hake and silver hake (upper right), Georges Bank and (spring only) Southern New England-Mid Atlantic yellowtail flounder (lower left). Ecoregions used in previous analyses are also shown (lower right). Further details, as well as the stock strata definition for white hake, are provided in S1 Supporting Information.

Distribution shifts in the Northwest Atlantic have been examined in a number of studies [6, 13–15]. Nye et al. [6] reported poleward shifts in the CG for 15 of 30 pelagic and groundfish species using spring Northeast Fisheries Science Center (NEFSC) bottom trawl survey data, 1968–2007. Analysis for some of the groundfish species, including Atlantic cod, red hake (*Urophycis chuss*), silver hake and yellowtail flounder (*Limanda ferruginea*), was divided into northern and southern “ecoregions.” However, these ecoregions are not congruent with the boundaries used for stock assessment and management of these species (Fig 1). An unresolved question has been to what extent the results of previous analyses might have differed if these stock boundaries had been used. Additionally, the effect of fishing pressure on fish spatial distribution in the Northwest Atlantic has been limited to the aforementioned study on silver hake [10] and another study that used changes in abundance and length-structure as an index of fishing pressure [14]. Thus, another ongoing question has been the role of catch on distribution shifts in the Northwest Atlantic.

There were two objectives to this study. The first objective was to reevaluate (1968–2007) and update (2008–2016) the spring spatiotemporal distribution of cod, red hake, silver hake, white hake (*Urophycis tenuis*) and yellowtail flounder using currently established stock boundaries. This work builds upon the previous analysis of Nye et al. [6] by adding nine years of data to the time series, and addresses to what extent stock boundaries and other minor methodological differences might impact results. The same analysis was also done for the fall (1963–2016) to determine whether trends can be extrapolated across seasons. The second objective of this study was to evaluate the relative importance of density-dependent, exploitation and temperature effects on spatial distribution with an information theoretic approach. Estimating the relative importance of variables with summed Akaike weights is an alternative to stepwise variable selection that considers model selection uncertainty [16]. The technique is popular in terrestrial ecology (see review in [17]) and has recently been used to gauge the relative importance of climate and other environmental indices on salmon migration and habitat [18–19]. In the present study, Akaike weights were used to evaluate the relative importance of population size, fishing pressure and bottom temperature on the spatial distribution of the groundfish described above.

## Materials and methods

### Data

The NEFSC has conducted spring and fall bottom trawl surveys on the continental shelf of the Northeast United States since 1968 and 1963, respectively (Fig 1). The survey employs a stratified random design. Strata are defined primarily by depth, and the number of stations allocated to each stratum is proportional to stratum area. Several gear and vessel changes have occurred over the course of the survey [20]. Conversion factors to account for these changes were applied as necessary. The stocks considered in this analysis are listed in Table 1. Bottom temperature data are collected at all NEFSC trawl stations [21]. Catch data (landings + discards) through 2016 were taken from the most recent assessment update for each stock (links are provided in S1 Supporting Information). A complete time series of catch data was available for all stocks except Gulf of Maine (GOM) cod, which begins in 1982.

**Table 1:** Northwest Atlantic groundfish stocks analyzed in the present study.

Common name	Scientific name	Stock	Acronym
Atlantic cod	*Gadus morhua*	Gulf of Maine	GOM
		Georges Bank	GB
Red hake	*Urophycis chuss*	Northern	Northern
		Southern	Southern
Silver hake	*Merluccius bilinearis*	Northern	Northern
		Southern	Southern
White hake	*Urophycis tenuis*	Unit	Unit
Yellowtail flounder	*Limanda ferruginea*	Georges Bank	GB
		Southern New England-Mid Atlantic	SNEMA

### Data preparation

Distances between points (trawl stations) were computed in a Euclidean reference system [22]. This was done by setting the minimum longitude and latitude of the strata for each stock as (0, 0) and converting all coordinates to kilometers. The cosine of the midpoint latitude for each stock was used to convert longitude. This process is also known as geographical referencing [23].

### Spatial indicators

The center of gravity (CG) is the bivariate mean location of the population [22, 24–26] (hereafter referred to as the X- and Y- components of the CG [27]):

$$CG=\frac{\sum_{i=1}^{n}x_{i}w_{i}z_{i}}{\sum_{i=1}^{n}w_{i}z_{i}}$$ (1)

where *x*_*i*_ (*i* = 1,…, *n*) is location (geographically referenced longitude or latitude), *w*_*i*_ is the area of influence, and *z*_*i*_ is the biomass of groundfish. In the case of irregular sampling, spatial indicators are weighted with an area of influence [22, 24–26]. Given the stratified random survey design (as opposed to a grid), a Dirichlet tessellation, also known as Voronoï polygons [28], was used as a non-subjective method to calculate areas of influence, with areas along the edge of the study area clipped to the boundary of the strata. Prior to analysis, the CG of sample locations (unweighted by *z*_*i*_) was calculated and regressed as a function of year to verify that changes in the CG over time were not due to changes in sampling design [26]. Biomass weighted mean depth was also calculated with Eq 1 by replacing location *x*_*i*_ with depth [29].

The inertia (variance) describes how dispersed the population is around its CG [22, 24–26]:

$$I=\frac{\sum_{i=1}^{n}{\left(x_{i}-CG\right)}^{2}w_{i}z_{i}}{\sum_{i=1}^{n}w_{i}z_{i}}$$ (2)

*I* can be decomposed into two orthogonal axes describing the maximum and the minimum components of the inertia. The square root of *I* for a given axis gives the standard deviation of the respective axis. As *I* has units of km^2^, axes of inertia are plotted on CG maps as the standard deviation, which has units of km [25–26].

The positive area (PA) is the area (km^2^) occupied by fish biomass greater than zero [25–26]:

$$PA=\sum_{i=1}^{n}w_{i}[z_{i}>0]$$ (3)

The PA is analogous to the proportion of positive tows, albeit weighted with the area of influence *w*_*i*_.

### Time series analysis of spatial distribution

The same methods were used for this analysis as in Nye et al. [6] to facilitate comparisons between the two studies. Briefly, the relationship between each spatial indicator and year was modeled with a linear regression (i.e., a generalized linear model with Gaussian distribution and identity link function). Models that exhibited serial correlation were corrected with a first order autoregressive fit [30]. Years in which not all strata were sampled for a particular stock were omitted from this analysis (Table 2). The impact of this on the data sets was generally negligible, except for the southern hake stocks. The latter was due, in part, to the fact that the southernmost strata were not added to the survey until fall 1967 [20].

**Table 2:** Sample sizes (*n*) for objective 1 (time series analysis of spatial distribution) and objective 2 (relative importance of predictor variables on spatial distribution).

		Objective 1		Objective 2	
Common Name	Stock	Spring (*n*)	Fall (*n*)	Spring (*n*)	Fall (*n*)
Atlantic cod	GOM	48	54	31	29
Atlantic cod	GB	47	54	37	41
Red hake	Northern	48	54	41	46
Red hake	Southern	39	40	20	21
Silver hake	Northern	48	54	41	46
Silver hake	Southern	37	40	20	21
White hake	Unit	48	54	41	46
Yellowtail flounder	GB	47	54	37	41
Yellowtail flounder	SNEMA	49	54	46	51

Northeast Fisheries Science Center bottom trawl survey data used in this analysis: spring, 1968–2016 (*n* = 49); and fall, 1963–2016 (*n* = 54). Years in which not all strata were sampled for a particular stock were omitted for objective 1. Additionally, years in which there were insufficient bottom temperature recordings to calculate a stratified mean that was representative of the entire stock area were omitted for objective 2. Sample size for GOM cod is further reduced because the times series of catch data begins in 1982. Stock acronyms: Gulf of Maine (GOM); Georges Bank (GB); and Southern New England-Mid Atlantic (SNEMA).

### Relative importance of predictor variables on spatial distribution

Three predictor variables were considered for this analysis: stratified mean kg per tow from the NEFSC survey (hereafter referred to as biomass) was used as an index of relative population size; while stratified mean bottom temperature (°C) from the NEFSC survey (hereafter temperature) was used as an index of temperature regime. Stratified mean surface temperature from the survey was collinear with bottom temperature in most cases and thus was not used in this analysis of groundfish. Catch divided by stratified mean kg per tow from the NEFSC survey (relative *F* [31]) was used as an index of fishing pressure. Other indices of fishing pressure were considered, but ultimately rejected, during the planning stages of this analysis: stock assessment model estimates of fishing mortality *F* were not used given the potential problems associated with treating stock assessment model output as “data” [32]; and CGs of reported catch (e.g., [11–12, 33–34]) were not used because of the known misreporting issues for some of the stocks considered here [35]. Biomass and relative *F* were log transformed. As with the time series analysis, years in which not all strata were sampled for a particular stock were omitted (Table 2). Additionally, years in which there were insufficient bottom temperature recordings to calculate a stratified mean that was representative of the entire stock area were omitted. As before, the impact of this on the data sets was greatest for the southern hake stocks (Table 2). Times series plots of predictor variables for each stock are provided in S1 Supporting Information.

The relative importance of predictor variables on spatial indices was evaluated by summing Akaike weights across all models in the set (Table 3) where predictor variable *j* occurs [16]. Akaike weights are defined as the relative likelihood of the model, given the data. Linear relationships between each spatial indicator and the predictor variables were modeled with generalized least squares [30] to allow for correlation in the error terms. Stocks were considered individually by season (rather than in a hierarchical mixed model) because it was hypothesized *a priori*, based on previous analyses (e.g. [6]), that predictors might have opposing effects on spatial distribution, e.g. stock *a* moves north in response to warming while stock *b* moves south. Following the recommendation of Arnold [17], a list of individual predictors, their cumulative model weights, and model averaged parameter estimates for each stock are provided in S1 Supporting Information.

**Table 3:** The set of models used to sum Akaike weights.

(intercept only)
Biomass
Relative *F*
Temperature
Biomass + Relative *F*
Biomass + Temperature
Relative *F* + Temperature
Biomass + Relative *F* + Temperature

### Software

Several R [36] packages were used in this analysis: spatial indicators were calculated in RGeostats [37]; Dirichlet tessellae were calculated in spatstat [38]; generalized least squares fits were modeled in nlme [39]; while the relative importance of predictors and model averaged predictor estimates were calculated with MuMIn [40]. Time series autoregressive fits were modeled in PROC AUTOREG (SAS Institute Inc., Cary, NC, USA).

## Results

The following results are a broad overview of findings for all the stocks considered in this analysis. Complete results for each stock can be found in S1 Supporting Information.

### Time series analysis of spatial distribution

In the spring, the CG for eight of nine stocks exhibited a significant northward and/or eastward shift over the course of time series (Table 4). GOM cod was the only stock to shift southward. The northward shift of northern silver hake was accompanied by an increase in inertia. In contrast, the eastward shift of Georges Bank (GB) yellowtail flounder, and the northeastward shift of Southern New England-Mid Atlantic (SNEMA) yellowtail flounder, were both accompanied by decreased dispersion around their respective CGs.

**Table 4:** Summary of time series trends in the spatial distribution of nine Northwest Atlantic groundfish stocks.

		Spring					Fall				
Common Name	Stock	XCG	YCG	Inertia	Depth	PA	XCG	YCG	Inertia	Depth	PA
Atlantic cod	GOM		-1.1		-1.0	-278	-1.2	-0.5		-0.9	-301
Atlantic cod	GB	1.0	0.7		0.5	-284					-349
Red hake	Northern	0.8			0.3	758	0.4			0.8	834
Red hake	Southern	6.2	1.8		1.0		2.7			0.5	
Silver hake	Northern		0.7	42	-0.6	606		0.8			206
Silver hake	Southern	5.6	2.4				2.9	0.8			
White hake	Unit	0.7					0.5			0.5	
Yellowtail flounder	GB	0.9		-57	0.2		1.2	0.6	-64		-209
Yellowtail flounder	SNEMA	1.2	0.8	-193	0.1	-665			-119	0.1	-443

Spatial indicators and associated units are: geographically referenced longitude and latitude of the center of gravity (XCG and YCG, respectively; km), inertia (km^2^), depth (m) and positive area (PA; km^2^). Only significant (*p* < 0.05) slopes are shown. For brevity, inertia and PA are rounded to the nearest integer. Stock acronyms: Gulf of Maine (GOM); Georges Bank (GB); and Southern New England-Mid Atlantic (SNEMA).

Five stocks shifted deeper over the course of the spring time series (Table 4), and three of these shifts (GB cod, southern red hake, SNEMA yellowtail flounder) were concurrent with a northeastward shift in the CG. The mean depth of GOM cod decreased as the stock moved southward, while the average depth of northern silver hake decreased as it shifted northward.

Both northern hake stocks increased area occupancy, indicating range expansions (Table 4). GOM cod experienced a range contraction as the CG shifted southward and shallower. GB cod and SNEMA yellowtail flounder also had range contractions as the CG for both stocks shifted northeastward and deeper.

Only two stocks exhibited the same changes in spatial distribution in the fall as compared with the spring (Table 4). The range expansion eastward and deeper for northern red hake was the same in spring and fall, while southern silver hake shifted northeastward in both seasons. All other stocks had at least one difference between seasons. For example, a westward component to the southward shift of GOM cod was detected in the fall. In contrast, the northeastward shift of southern red hake in the spring was reduced in the fall, such that the northward component was no longer significant.

### Relative importance of predictor variables on spatial distribution

**Fig 2:**
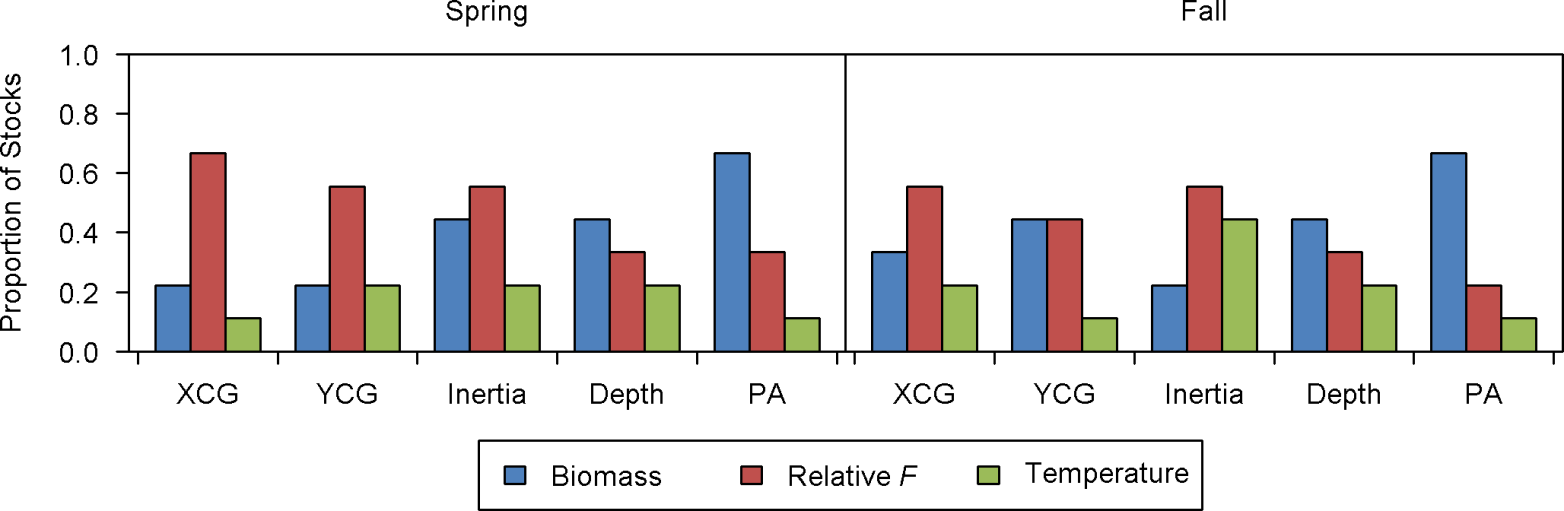
Proportion of stocks for which a variable was the most important predictor, by spatial indicator. Spatial indicators and associated units are: geographically referenced longitude and latitude of the center of gravity (XCG and YCG, respectively; km), inertia (km^2^), depth (m) and positive area (PA; km^2^). Predictor variables are: Northeast Fisheries Science Center (NEFSC) bottom trawl survey stratified mean kg per tow (biomass; kg), catch/NEFSC stratified mean kg per tow (relative *F*) and NEFSC stratified mean bottom temperature (°C). Proportions may not sum to one in the case of ties.

Patterns of relative importance in the spring were dominated by relative *F* and biomass (Fig 2). Relative *F* ranked first for 67% of stocks in terms of the XCG, and five of nine stocks for both the YCG and inertia. The index of fishing pressure was the most important predictor of the CG and associated inertia for GOM cod, GB cod, and GB yellowtail flounder (S1 Supporting Information).

Biomass ranked first for four stocks in terms of depth, and 67% of stocks for area occupancy (Fig 2). Biomass was the most important predictor of all spatial indices for SNEMA yellowtail flounder (S1 Supporting Information), the only case where a single predictor ranked first for all indices in either season.

Patterns of relative importance in the fall were also dominated by relative *F* and biomass (Fig 2). Relative *F* ranked first for five of nine stocks in terms of the XCG and inertia. The index of fishing pressure also ranked first for the YCG for four stocks, the same number of cases as biomass. GB cod was the only stock for which relative *F* was the most important predictor of the CG and associated inertia across seasons (S1 Supporting Information).

As in the spring, biomass ranked first for four stocks in terms of depth in the fall, and 67% of stocks for area occupancy. Biomass was the most important predictor of depth for both northern hake stocks across seasons (S1 Supporting Information). Similarly, biomass was the most important predictor of area occupancy in both seasons for five stocks: GOM cod, GB cod, southern silver hake, GB yellowtail flounder and SNEMA yellowtail flounder.

Temperature ranked first in the fewest number of cases in spring or fall (Fig 2). Most notably, it was not ranked first as a predictor of any spatial indicator for GOM cod in either season (S1 Supporting Information). In contrast, temperature ranked first as a predictor of four spatial indices (all but XCG) for white hake in the spring.

## Discussion

The first objective of this study was to reevaluate and update the spring spatiotemporal distribution of nine Northwest Atlantic groundfish stocks using survey strata consistent with the stock assessments, and to provide a similar time series analysis for the fall. Northward movement in the spring was generally consistent with prior analyses, whereas changes in depth distribution and area occupancy were not [6]. Only two stocks (northern red hake and southern silver hake) exhibited the same changes in spatiotemporal distribution in the fall as compared with the spring. The second objective of this study was to evaluate the relative importance of population size, fishing pressure and bottom temperature on spatial distribution. Fishing pressure was the most important predictor of the bivariate mean location of the population for the majority of stocks in the spring, whereas in the fall this was restricted to the east-west component. Similarly, fishing pressure was the most important predictor of the dispersion around the mean location of the population in both spring and fall. Biomass was the most important predictor of area occupancy for the majority of stocks in both seasons.

### Time series analysis of spatial distribution

Northward movement in the spring for five of nine stocks is roughly consistent with the distribution shifts reported by Nye et al. [6]. Specifically, GB cod, southern red hake, and both silver hake stocks continue to show a northward shift in distribution; while it is unclear whether the poleward movement reported previously for southern yellowtail flounder continues, given that the southern ecoregion combines the GB and SNEMA stocks (Fig 1). The two clear differences are that the poleward movement reported previously for both northern red hake and white hake is no longer significant in the present analysis. This does not appear to be due to the additional years of data (S1 Supporting Information), and thus is likely due to other methodological differences between the two studies. In the previous analysis an along-shelf measure was used that follows the 200 m bathymetry contour to avoid CGs that are off the shelf [6]. However, it is a known property of the CG that fish may not be present at the CG location, that the CG may be on land, etc. [25–26]. Thus we adopted the standard geostatistical practice of calculating the CG in the Euclidean space of geographically referenced longitude and latitude [22–23]. This enables the calculation of a variance (i.e., inertia) that can be decomposed into two orthogonal axes describing the maximum and the minimum components of the inertia [22, 25–26]. One concern with the along-shelf measure in the Northwest Atlantic region is that, between 67°W and 71°W, “poleward” movement is essentially east-west (Fig 1). This illustrates another advantage of working in Euclidean space: the CG can be described in straightforward east-west (XCG) and north-south (YCG) units (km). Not surprisingly, recently developed methods for model-based estimates of the CG also adopt this approach [41].

Changes in spring depth distribution and area occupancy were less consistent with the analysis of Nye et al. [6]. Only one stock (southern red hake) was found to have shifted deeper in both studies. This is surprising, given the nearly identical formulation (Eq 1) of the biomass weighted depth index in the two studies. In terms of area occupancy, only two stocks (northern red hake, northern silver hake) exhibited an increase in both studies, while one stock (GB cod) decreased. This is not unexpected given that the area occupancy measures used in the two studies were quite different.

Analysis of the fall data revealed that only two of nine stocks exhibited the same trends in spatial distribution as in the spring: northern red hake increased area occupancy while shifting eastward and deeper in both seasons; and southern silver hake shifted northeast in both seasons. This illustrates that extrapolating results from one season to another should be done with caution. While this may seem intuitive it is nevertheless important to document as not all regions of the world have dedicated seasonal surveys. Other studies with access to intra-annual data have also found seasonal differences in the spatial distribution and prey consumption of cod and other groundfish in the Celtic Sea [42].

**Fig 3:**
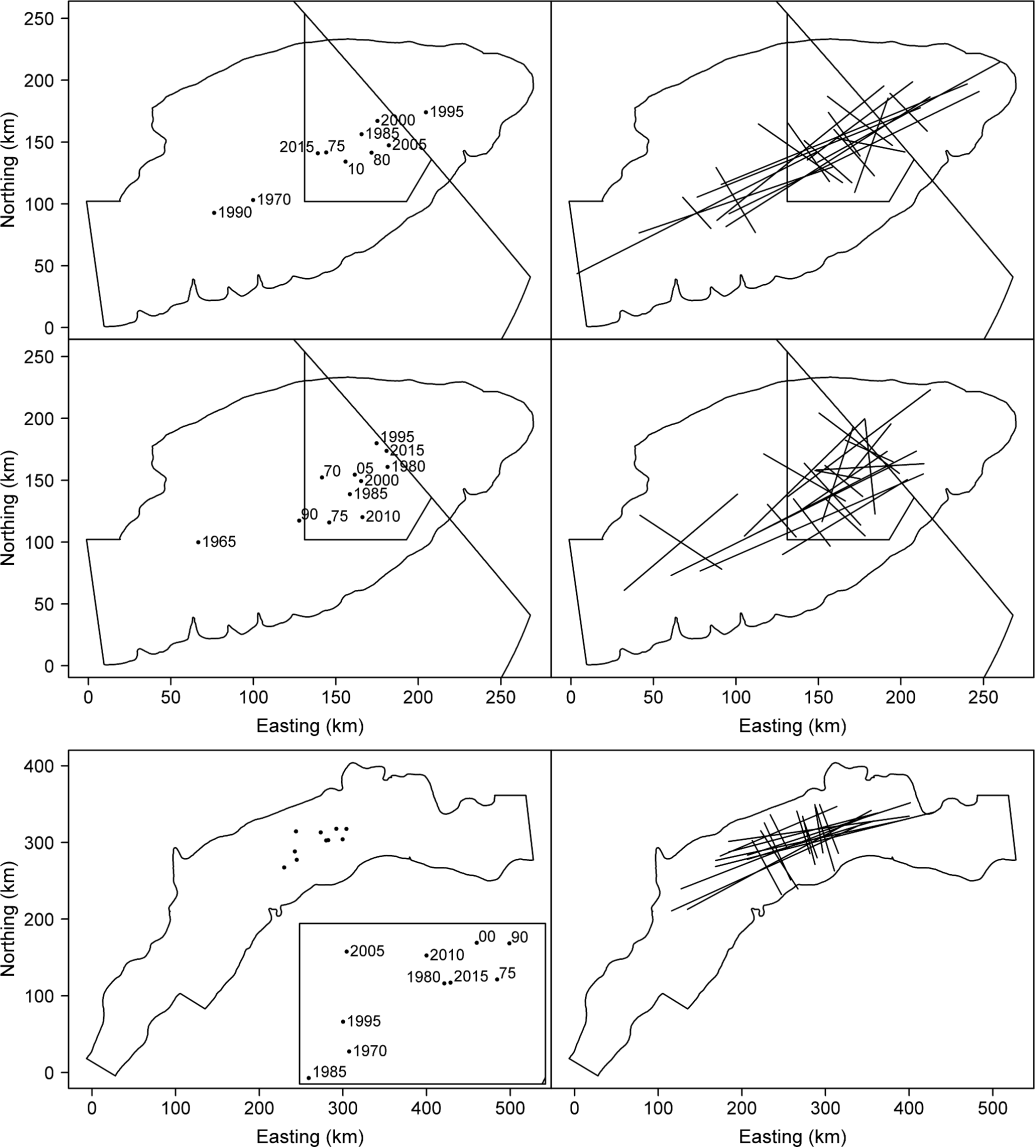
Maps for the three cases in this study where a significant change in the center of gravity was accompanied by a significant decrease in inertia. Centers of gravity (left) and inertia (right) for Georges Bank yellowtail flounder at five year intervals in the spring, 1970–2015 (upper), and in the fall, 1965–2015 (middle); and Southern New England-Mid Atlantic yellowtail flounder in the spring, 1970–2015 (lower). Closed Area II and the Hague Line are also shown in the upper and middle panels. Due to the tight grouping of the centers of gravity in the lower left panel the year labels are shown in the inset plot. Year labels for both stocks are truncated where necessary for clarity.

More complicated changes in spatial distribution can be revealed by considering two spatial indicators together. For example, the basin hypothesis (i.e., covariation of population density and area occupancy with biomass) of MacCall [43] can be observed as concurrent increases or decreases in the inertia and PA. Decreases in these two indices were observed for GB yellowtail flounder in the fall, and SNEMA yellowtail flounder in both seasons. Management concerns regarding shifts in distribution from one stock area to another (e.g. [44]) can also be addressed by considering two spatial indicators together. Such a distribution shift would be manifested as a change in one or both planes of the CG, along with a decrease in inertia as the stock approaches and moves across the boundary. This combination of trends in spatial indicators was only observed in three cases: GB yellowtail flounder in both spring and fall; and SNEMA yellowtail flounder in the spring. Fig 3 shows that neither stock appears to be shifting outside its respective stocks boundaries.

### Relative importance of predictor variables on spatial distribution

Fishing pressure was the most important predictor of the CG for the majority of stocks in the spring, whereas in the fall this was restricted to the east-west component. Fishing pressure was also the most important predictor of the dispersion around the CG in both spring and fall. While climate indices were not considered in this study, our results clearly show that fishing pressure, in addition to the previously established role of climate [6], affects the spatial distribution of groundfish in the Northwest Atlantic. In the North Sea both fishing mortality and climate have an effect on the CG and depth indices for sole [11]. Similarly, the northward and deepening shift of North Sea cod during the period 1913 to 2012 is attributable to warming, while the eastward component of the movement is best explained by fishing pressure [12]. More broadly, the demersal fish community in the North Sea has changed over the course of the 20th and 21st centuries in response to both climate and fishing pressure [45]. Finally, synergistic effects have also been shown, such that fishing pressure increases the climatic sensitivity of larval fishes in the California Current ecosystem [46].

Biomass was the most important predictor of area occupancy for both cod stocks, southern silver hake, and both yellowtail flounder stocks in spring and fall. This confirms previous analyses that have shown similar relationships for cod and yellowtail flounder in Canadian waters [47–49], as well as cod, American plaice (*Hippoglossoides platessoides*) and Greenland halibut (*Reinhardtius hippoglossoides*) on the Flemish Cap [50]. All of these findings comport with a recent analysis of six marine regions around the world which found that abundance-area occupancy relationships are strongest for Gadiformes, followed by Pleuronectiformes [51].

Bottom temperature was the most important predictor in the fewest number of cases. This general lack of a temperature effect is consistent with Nye et al. [6], who found that distributional shifts in the Northwest Atlantic were associated with large scale warming and climatic conditions, such as the Atlantic Multidecadal Oscillation, rather than annual temperature measures *per se*. Similarly, the majority of variation in the CG, including a northwest shift in distribution, for walleye pollock (*Gadus chalcogrammus*) in the Bering Sea is largely unexplained by bottom temperature [52].

### Future work

Two unresolved issues in this study suggest avenues for future analyses. First, a plausible mechanism for the relationship between relative *F* and spatial distribution needs to be elucidated. For example, if fishing were concentrated in the south of the stock distribution, this would result in a shift in the overall stock distribution to the north. However, fishing is not evenly distributed across the stock distribution for all sorts of reasons such as density of fish, distance from port, bycatch of other species, etc. Thus, an increase in *F* should result in a change in distribution, although one could probably not predict *a priori* how the distribution would change unless the fishery was specifically focused on only one region. The second unresolved question is the reliability of the PA as an area occupancy measure, as a zero may represent a low probability of capture rather than true absence from this area [47]. Thus the relationships found in this study should be verified with alternative area occupancy measures such as the minimum area containing a specified percentage of population biomass.

### Conclusions

This study can inform future assessments in several ways. First, while species level or ecoregion analyses may be of ecological interest, spatial distribution analyses intended to inform stock assessment or management should be based on spatial boundaries consistent with those used in the stock assessment. All stocks in this analysis had at least one spatial indicator that was different in terms of magnitude or direction than previous analyses that did not use stock boundaries. This is a general principle that extends beyond stock assessment and management of groundfish under United States jurisdiction in the Northwest Atlantic. Second, very few of the stock assessment packages currently used in the United States can represent spatial structure explicitly [53]. Should future advancements enable the incorporation of spatial indicators as data inputs, such indices must be based on stock boundaries, just as the survey indices that are used to tune catch data are. Finally, future spatial distribution analyses for a particular stock should be age-based, as ontogenetic changes may be masked when the data are not disaggregated by age [27]. This was not done in the present study because the goal was to examine trends across multiple stocks, rather than the spatiotemporal distribution of one stock in detail.

This study shows that fishing pressure, in addition to the previously established role of climate [6], influences the spatial distribution of groundfish in the Northwest Atlantic. More broadly, this study is one of a small but growing body of literature to demonstrate that fishing pressure, in addition to climate, has an effect on the spatial distribution of marine resources. It has recently been shown that fishing pressure can cause synchronous changes in stock abundance at spatial and temporal scales comparable to those attributed to climate forcing [54]. Altogether this mounting body of evidence necessitates that future work must consider both fishing pressure and climate when examining mechanisms underlying fish distribution shifts.

## Supporting information

S1 Supporting Information (PDF)

S1 Dataset Survey. (CSV)

S2 Dataset Catch. (CSV)

S1 Text Data field definitions. (TXT)
