# Supplementary material for: Relative importance of population size, fishing pressure and temperature on the spatial distribution of nine Northwest Atlantic groundfish stocks
Source: PLoS One. 2018 Apr 26;13(4):e0196583. doi: 10.1371/journal.pone.0196583 (PMC5919506; doi:10.1371/journal.pone.0196583)
Supplement: S1 Supporting Information — (PDF) [file pone.0196583.s001.pdf]

S1 Supporting Information. Stock Summaries

Table of Contents

Atlantic Cod (Georges Bank) 2

Atlantic Cod (Gulf of Maine) 6

Red Hake (Northern) 10

Red Hake (Southern) 14

Silver Hake (Northern) 18

Silver Hake (Southern) 22

White Hake (Unit) 26

Yellowtail Flounder (Georges Bank) 30

Yellowtail Flounder (Southern New England-Mid Atlantic) 34

Species: Atlantic Cod (*Gadus morhua*)
Stock: Georges Bank

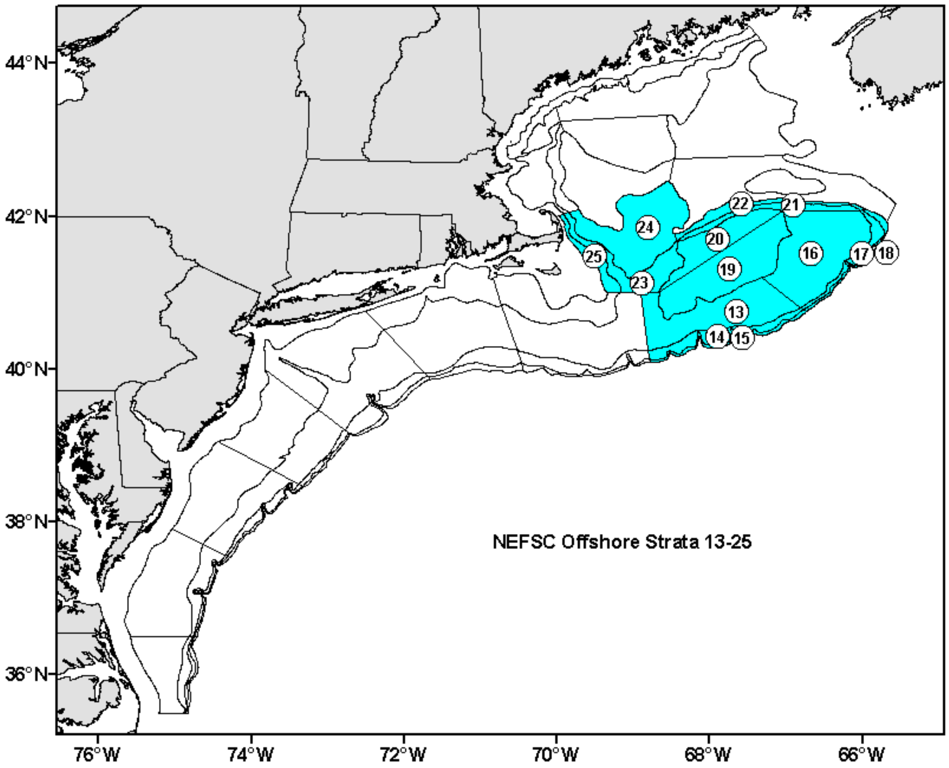

Northeast Fisheries Science Center bottom trawl survey offshore strata on the continental shelf of the Northeast United States. Atlantic cod (Georges Bank) stock strata are shown in blue.

NEFSC surveys used in the most recent assessment: Spring & Fall

Link to most recent benchmark assessment:
Northeast Fisheries Science Center. 55th Northeast regional stock assessment workshop (55th SAW) *assessment report*. US Dept Commer, Northeast Fish Sci Cent Ref Doc. 2013; 13-11. 845 pp. Available from: http://nefsc.noaa.gov/publications/crd/crd1311/.

Link to most recent assessment update:
Northeast Fisheries Science Center. Operational assessment of 19 Northeast groundfish stocks, updated through 2016. US Dept Commer, Northeast Fish Sci Cent Ref Doc. 2017; 17-17. 259 pp. Available from: http://www.nefsc.noaa.gov/publications/crd/crd1717/.

Time-series analysis

Estimate, standard error, *t*-value and *p*-value for linear regressions of Atlantic cod (Georges Bank) center of gravity (XCG, YCG), inertia, depth and positive area (PA) as a function of year. *P*-values < 0.05 are in bold. Corresponding time series plots are shown below, with significant linear fits indicated by a solid line. Sample sizes: spring (*n* = 47); fall (*n* = 54).

	Spring				Fall			
	Estimate	SE	*t*-value	*p*-value	Estimate	SE	*t*-value	*p*-value
XCG	1.00	0.23	4.28	**< 0.001**	0.31	0.37	0.85	0.399
YCG	0.66	0.14	4.59	**< 0.001**	0.17	0.12	1.48	0.146
Inertia	-3.34	27.55	-0.12	0.904	51.47	27.95	1.84	0.071
Depth	0.47	0.12	4.02	**< 0.001**	-0.17	0.11	-1.52	0.134
PA	-284.19	50.38	-5.64	**< 0.001**	-349.35	32.18	-10.86	**< 0.001**

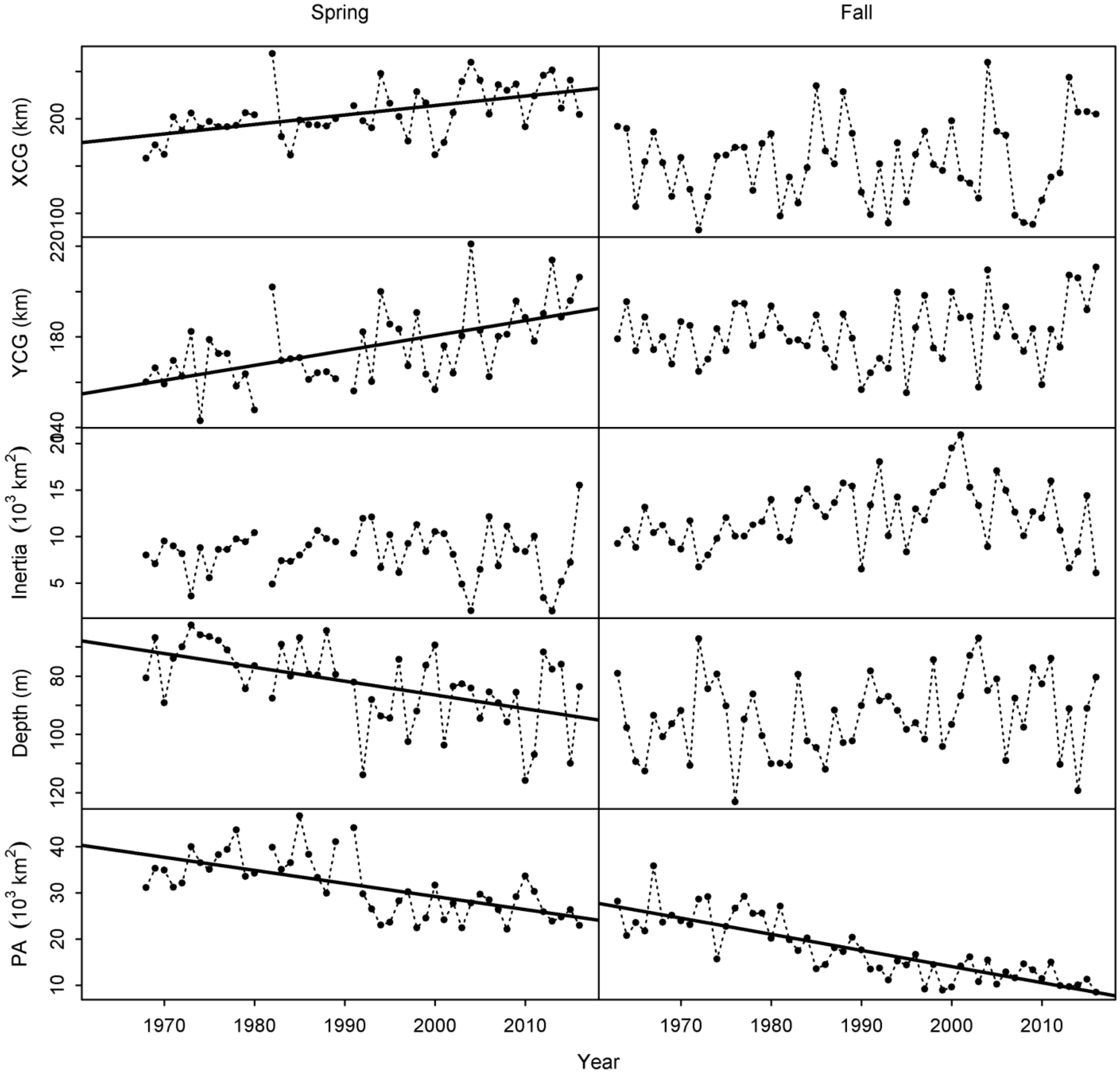

Predictor variables

Time-series of predictor variables for Atlantic cod (Georges Bank). Predictors and associated units are: Northeast Fisheries Science Center (NEFSC) bottom trawl survey stratified mean kg per tow (biomass), catch/NEFSC stratified mean kg per tow (relative *F*) and NEFSC stratified mean bottom temperature (°C). Data for all three predictors were set to NA for years in which there were insufficient bottom temperature recordings to calculate a stratified mean. Sample sizes: spring (*n* = 37); fall (*n* = 41).

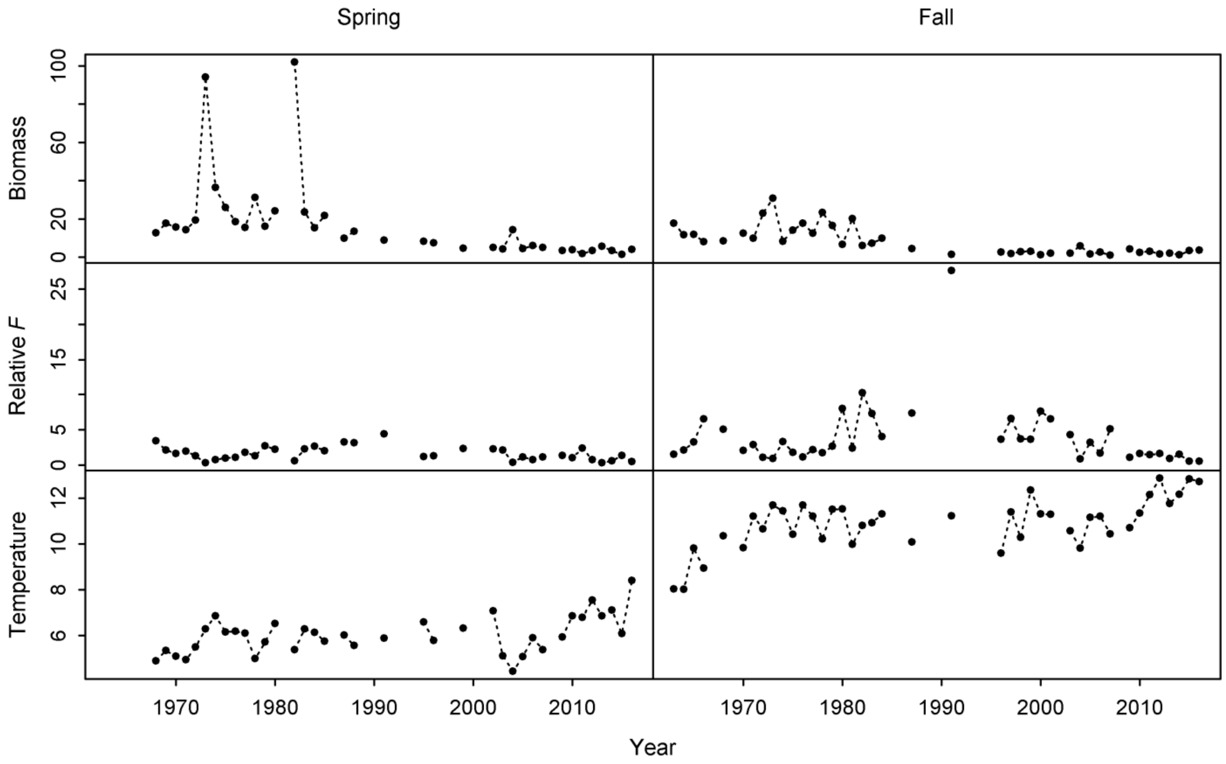

Relative importance of predictors on spatial distribution

Relative importance of predictor variables on spatial indicators for Atlantic cod (Georges Bank). Spatial indicators and associated units are: geographically referenced longitude and latitude of the center of gravity (XCG and YCG, respectively; km), inertia (km^2), depth (m) and positive area (PA; km^2). Predictor variables are: Northeast Fisheries Science Center (NEFSC) bottom trawl survey stratified mean kg per tow (biomass; kg), catch/NEFSC stratified mean kg per tow (relative *F*) and NEFSC stratified mean bottom temperature (°C). Predictor variables with the highest summed Akaike weights for each indicator are in bold.

	**Spring**			**Fall**		
Predictor	Biomass	Relative *F*	Temperature	Biomass	Relative *F*	Temperature
XCG	0.57	**0.88**	0.71	0.40	**0.67**	0.23
YCG	0.89	**1.00**	0.54	0.44	**0.75**	0.24
Inertia	0.22	**0.91**	**0.91**	0.59	**0.87**	0.26
Depth	**0.99**	0.21	0.34	0.28	**0.29**	0.28
PA	**0.99**	0.76	0.23	**1.00**	0.91	0.41

Model-averaged parameter estimates

Model-averaged predictor estimates by spatial indicator for Atlantic cod (Georges Bank). Spatial indicators and associated units are: geographically referenced longitude and latitude of the center of gravity (XCG and YCG, respectively; km), inertia (km^2), depth (m) and positive area (PA; km^2). Predictor variables are: Northeast Fisheries Science Center (NEFSC) bottom trawl survey stratified mean kg per tow (biomass; kg), catch/NEFSC stratified mean kg per tow (relative *F*) and NEFSC stratified mean bottom temperature (°C).

	**Spring**			**Fall**		
	Biomass	Relative *F*	Temperature	Biomass	Relative *F*	Temperature
XCG	-5.41	-16.07	-9.01	-4.27	-10.94	-0.21
YCG	-6.28	-19.46	-2.77	-1.35	-3.85	0.15
Inertia	5.45	1591.91	1447.26	-689.10	1380.98	77.63
Depth	-8.05	-0.18	-0.83	0.54	0.68	0.50
PA	4656.29	2009.67	-120.85	6667.70	1219.72	-225.40

Species: Atlantic Cod (*Gadus morhua*)
Stock: Gulf of Maine

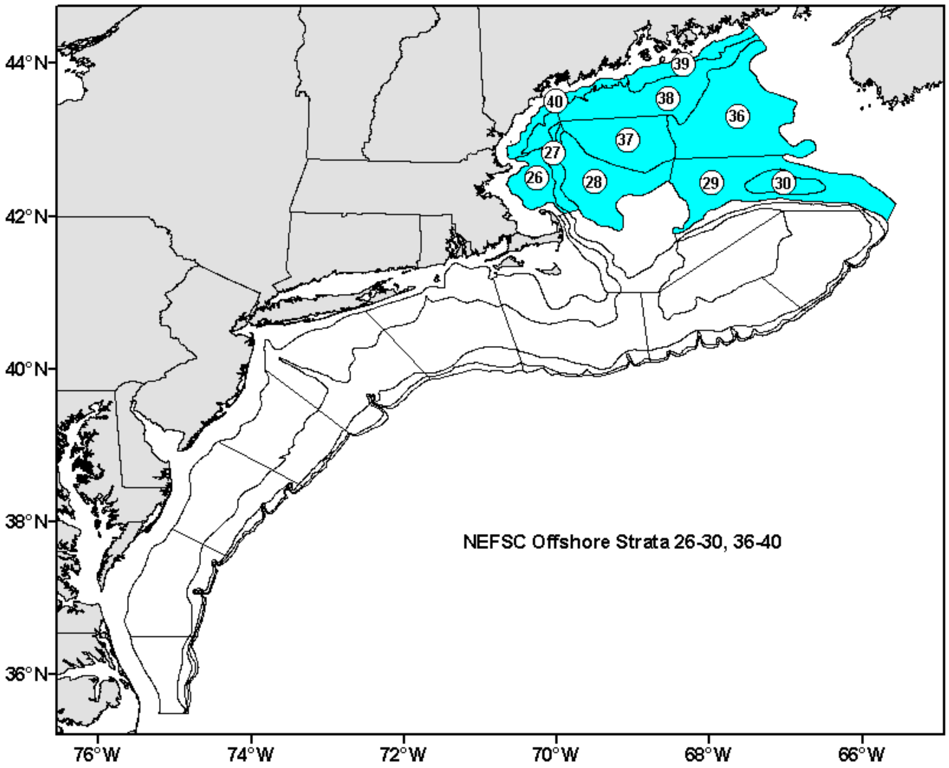

Northeast Fisheries Science Center bottom trawl survey offshore strata on the continental shelf of the Northeast United States. Atlantic cod (Gulf of Maine) stock strata are shown in blue.

NEFSC surveys used in the most recent assessment: Spring & Fall

Link to most recent benchmark assessment:
Northeast Fisheries Science Center. 55th Northeast regional stock assessment workshop (55th SAW) *assessment report*. US Dept Commer, Northeast Fish Sci Cent Ref Doc. 2013; 13-11. 845 pp. Available from: http://nefsc.noaa.gov/publications/crd/crd1311/.

Link to most recent assessment update:
Northeast Fisheries Science Center. Operational assessment of 19 Northeast groundfish stocks, updated through 2016. US Dept Commer, Northeast Fish Sci Cent Ref Doc. 2017; 17-17. 259 pp. Available from: http://www.nefsc.noaa.gov/publications/crd/crd1717/.

Time-series analysis

Estimate, standard error, *t*-value and *p*-value for linear regressions of Atlantic cod (Gulf of Maine) center of gravity (XCG, YCG), inertia, depth and positive area (PA) as a function of year. *P*-values < 0.05 are in bold. Corresponding time series plots are shown below, with significant linear fits indicated by a solid line. Sample sizes: spring (n = 48); fall (n = 54).

	Spring				Fall			
	Estimate	SE	*t*-value	*p*-value	Estimate	SE	*t*-value	*p*-value
XCG	-0.75	0.45	-1.67	0.101	-1.20	0.48	-2.50	**0.016**
YCG	-1.11	0.13	-8.33	**< 0.001**	-0.46	0.15	-3.02	**0.004**
Inertia	-39.96	46.71	-0.86	0.397	-44.49	35.64	-1.25	0.218
Depth	-1.01	0.42	-2.43	**0.019**	-0.87	0.25	-3.54	**< 0.001**
PA	-277.70	77.96	-3.56	**< 0.001**	-300.80	67.91	-4.43	**< 0.001**

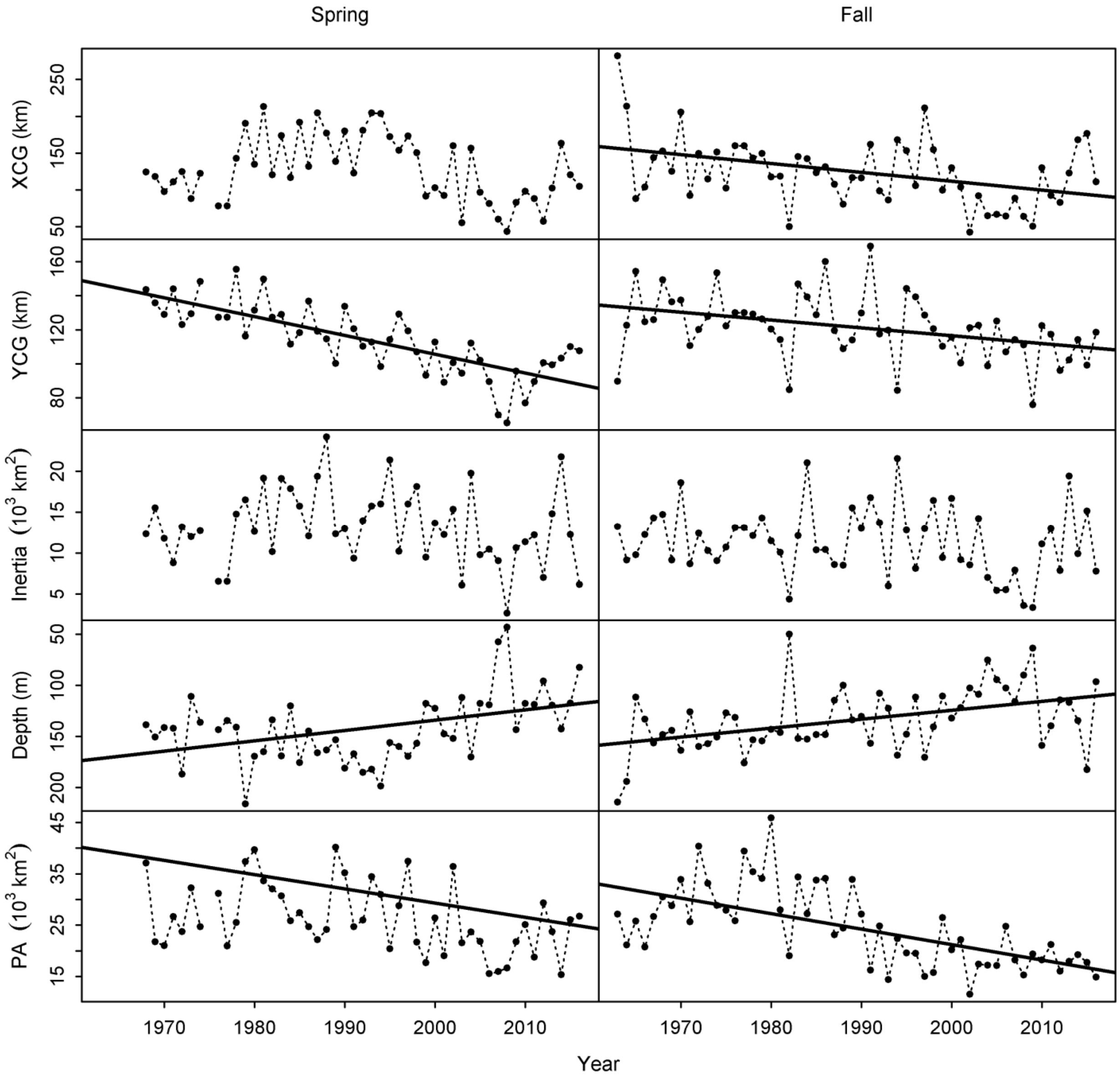

Predictor variables

Time-series of predictor variables for Atlantic cod (Gulf of Maine). Predictors and associated units are: Northeast Fisheries Science Center (NEFSC) bottom trawl survey stratified mean kg per tow (biomass), catch/NEFSC stratified mean kg per tow (relative *F*) and NEFSC stratified mean bottom temperature (°C). Catch data in the most recent assessment (NEFSC, 2013) begins in 1982, so data for all three predictors were set to NA for years prior. Additionally, data for all three predictors were set to NA for years in which there were insufficient bottom temperature recordings to calculate a stratified mean. Sample sizes: spring (*n* = 31); fall (*n* = 29).

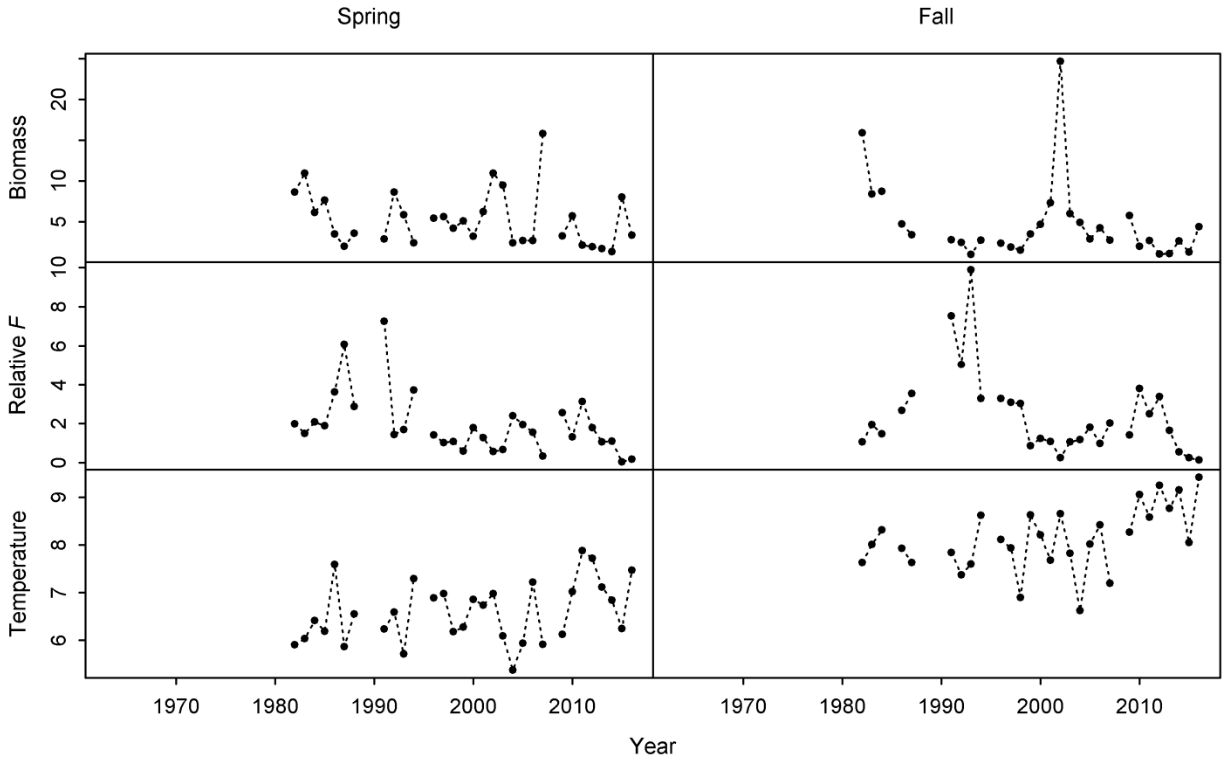

Relative importance of predictors on spatial distribution

Relative importance of predictor variables on spatial indicators for Atlantic cod (Gulf of Maine). Spatial indicators and associated units are: geographically referenced longitude and latitude of the center of gravity (XCG and YCG, respectively; km), inertia (km^2), depth (m) and positive area (PA; km^2). Predictor variables are: Northeast Fisheries Science Center (NEFSC) bottom trawl survey stratified mean kg per tow (biomass; kg), catch/NEFSC stratified mean kg per tow (relative *F*) and NEFSC stratified mean bottom temperature (°C). Predictor variables with the highest summed Akaike weights for each indicator are in bold.

	**Spring**			**Fall**		
	Biomass	Relative *F*	Temperature	Biomass	Relative *F*	Temperature
XCG	0.22	**0.34**	0.32	**0.68**	0.23	0.21
YCG	0.32	**0.37**	0.20	0.25	**0.49**	0.24
Inertia	0.30	**0.44**	0.41	**0.31**	0.24	0.22
Depth	0.26	**0.84**	0.30	**0.68**	0.25	0.26
PA	**0.52**	0.24	0.24	**0.39**	0.30	0.20

Model-averaged parameter estimates

Model-averaged predictor estimates by spatial indicator for Atlantic cod (Gulf of Maine). Spatial indicators and associated units are: geographically referenced longitude and latitude of the center of gravity (XCG and YCG, respectively; km), inertia (km^2), depth (m) and positive area (PA; km^2). Predictor variables are: Northeast Fisheries Science Center (NEFSC) bottom trawl survey stratified mean kg per tow (biomass; kg), catch/NEFSC stratified mean kg per tow (relative *F*) and NEFSC stratified mean bottom temperature (°C).

	**Spring**			**Fall**		
	Biomass	Relative *F*	Temperature	Biomass	Relative *F*	Temperature
XCG	-0.80	3.71	-4.29	15.37	-1.16	-1.12
YCG	-1.22	1.26	-0.05	-0.93	-3.11	1.22
Inertia	-417.07	549.07	-761.45	355.58	-154.02	-172.54
Depth	0.59	12.87	-2.44	-10.05	1.18	1.96
PA	1577.41	120.90	274.31	785.95	361.13	17.41

Species: Red Hake (*Urophycis chuss*)
Stock: Northern

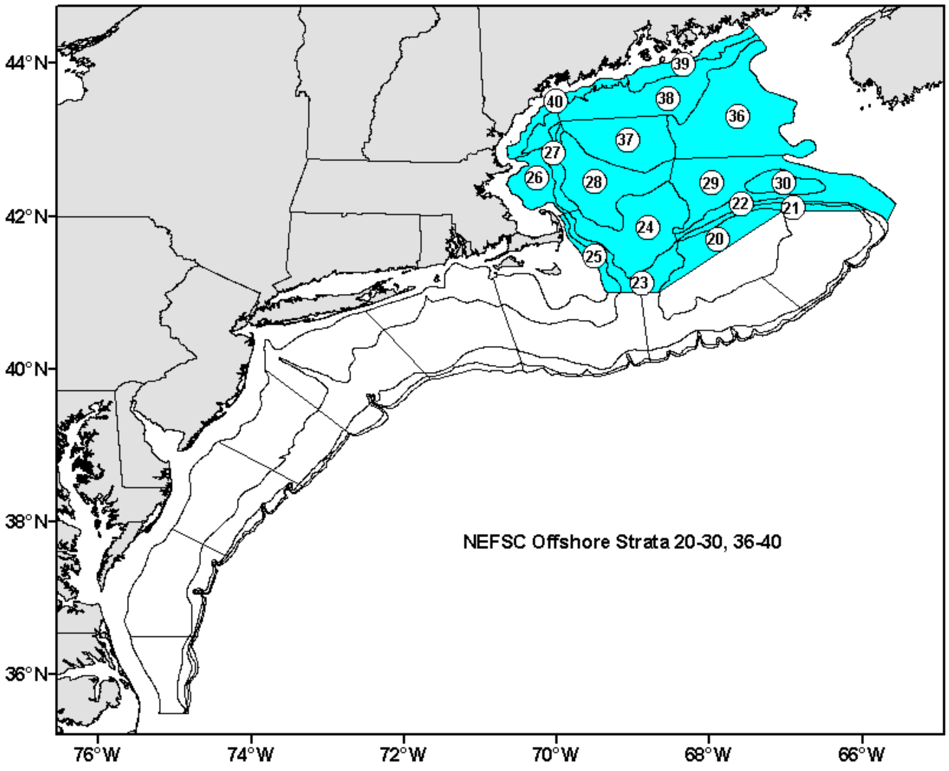

Northeast Fisheries Science Center bottom trawl survey offshore strata on the continental shelf of the Northeast United States. Red hake (northern) stock strata are shown in blue.

NEFSC surveys used in the most recent assessment: Spring only

Link to most recent benchmark assessment:

Northeast Fisheries Science Center. 51st Northeast regional stock assessment workshop (51st SAW) *assessment report*. US Dept Commer, Northeast Fish Sci Cent Ref Doc. 2011; 11-02. 856 pp. Available from: http://www.nefsc.noaa.gov/publications/crd/crd1102/.

Link to most recent assessment update:

New England Fisheries Management Council. Small-mesh multispecies fishing year 2016-2017 specifications supplemental information report (SIR), and regulatory flexibility analysis (RFA). 2016. 33 pp. Available from: http://s3.amazonaws.com/nefmc.org/2016-2017-Specifications-Supplemental-Information-Report.pdf.

Time-series analysis

Estimate, standard error, *t*-value and *p*-value for linear regressions of red hake (northern) center of gravity (XCG, YCG), inertia, depth and positive area (PA) as a function of year. *P*-values < 0.05 are in bold. Corresponding time series plots are shown below, with significant linear fits indicated by a solid line. Sample sizes: spring (*n* = 48); fall (*n* = 54).

	Spring				Fall			
	Estimate	SE	*t*-value	*p*-value	Estimate	SE	*t*-value	*p*-value
XCG	0.81	0.16	5.18	**< 0.001**	0.42	0.15	2.74	**0.008**
YCG	0.13	0.15	0.90	0.375	-0.13	0.23	-0.59	0.558
Inertia	9.26	12.72	0.73	0.470	-34.64	18.63	-1.86	0.069
Depth	0.34	0.14	2.50	**0.016**	0.82	0.21	3.91	**< 0.001**
PA	758.00	84.19	9.00	**< 0.001**	833.72	135.51	6.15	**< 0.001**

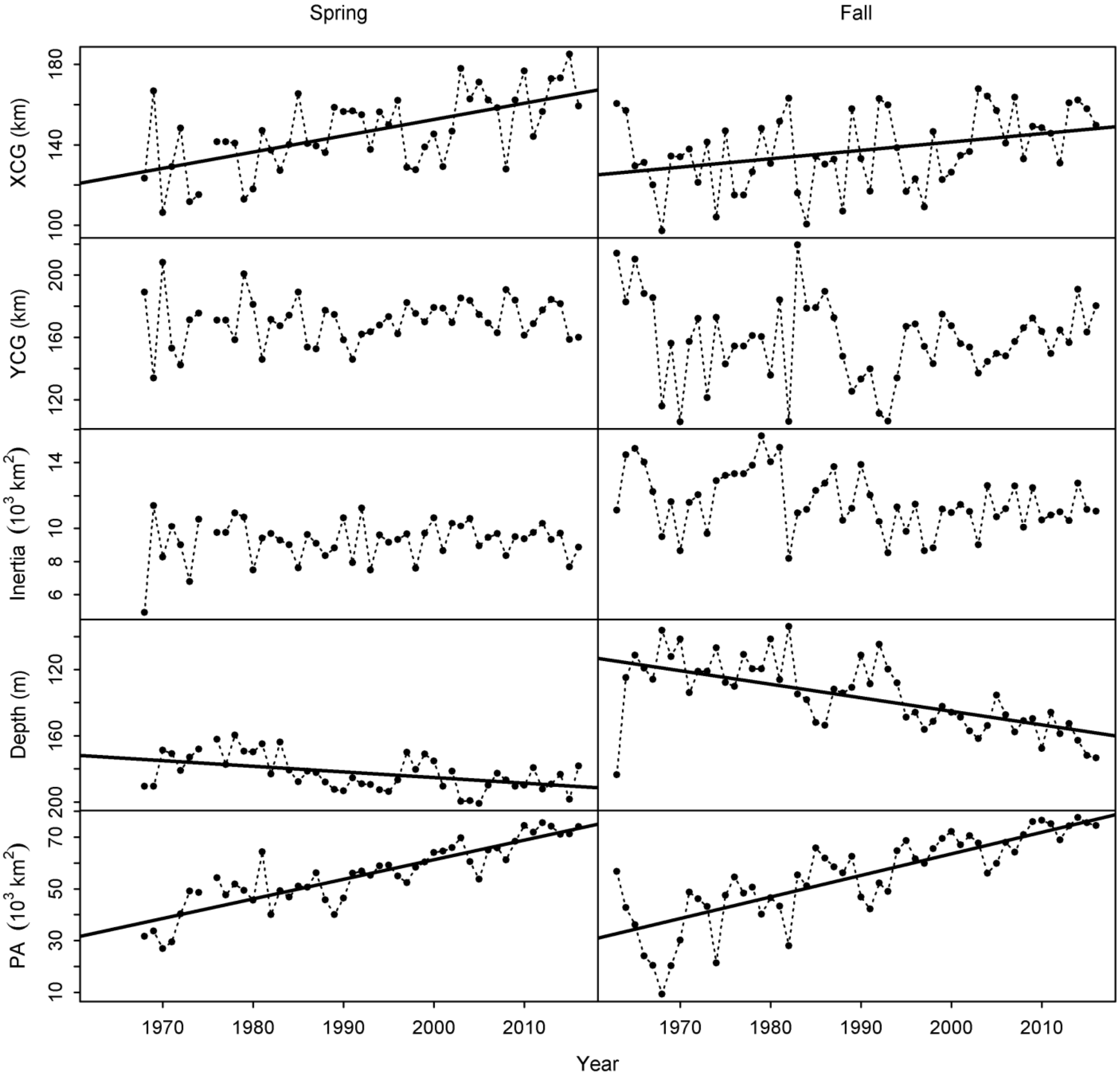

Predictor variables

Time-series of predictor variables for red hake (northern). Predictors and associated units are: Northeast Fisheries Science Center (NEFSC) bottom trawl survey stratified mean kg per tow (biomass), catch/NEFSC stratified mean kg per tow (relative *F*) and NEFSC stratified mean bottom temperature (°C). Data for all three predictors were set to NA for years in which there were insufficient bottom temperature recordings to calculate a stratified mean. Sample sizes: spring (n = 41); fall (n = 46).

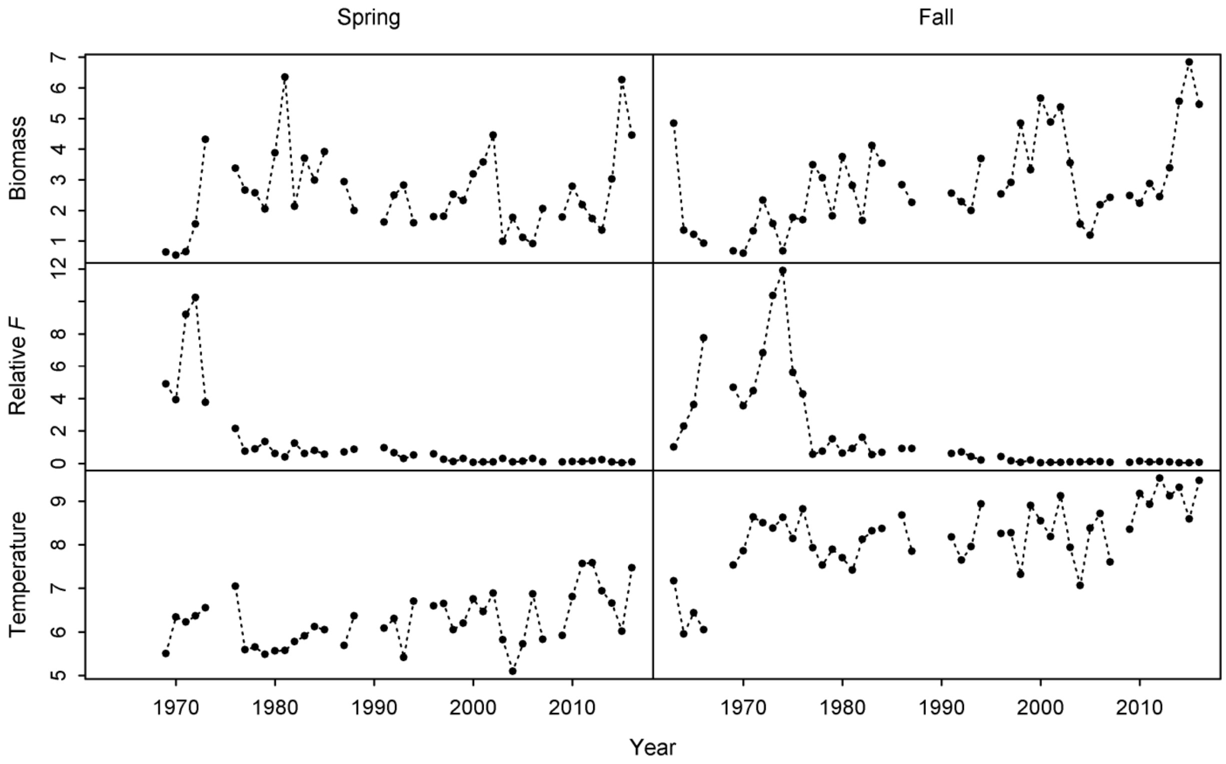

Relative importance of predictors on spatial distribution

Relative importance of predictor variables on spatial indicators for red hake (northern). Spatial indicators and associated units are: geographically referenced longitude and latitude of the center of gravity (XCG and YCG, respectively; km), inertia (km^2), depth (m) and positive area (PA; km^2). Predictor variables are: Northeast Fisheries Science Center (NEFSC) bottom trawl survey stratified mean kg per tow (biomass; kg), catch/NEFSC stratified mean kg per tow (relative *F*) and NEFSC stratified mean bottom temperature (°C). Predictor variables with the highest summed Akaike weights for each indicator are in bold.

	**Spring**			**Fall**		
Predictor	Biomass	Relative *F*	Temperature	Biomass	Relative *F*	Temperature
XCG	0.43	**0.67**	0.22	0.29	**0.76**	**0.76**
YCG	0.32	**0.61**	0.22	**0.57**	0.35	0.26
Inertia	**0.77**	0.35	0.22	0.25	0.44	**0.51**
Depth	**0.82**	0.71	0.48	**0.90**	0.41	0.37
PA	0.33	**0.99**	0.97	**0.99**	0.72	0.89

Model-averaged parameter estimates

Model-averaged predictor estimates by spatial indicator for red hake (northern). Spatial indicators and associated units are: geographically referenced longitude and latitude of the center of gravity (XCG and YCG, respectively; km), inertia (km^2), depth (m) and positive area (PA; km^2). Predictor variables are: Northeast Fisheries Science Center (NEFSC) bottom trawl survey stratified mean kg per tow (biomass; kg), catch/NEFSC stratified mean kg per tow (relative *F*) and NEFSC stratified mean bottom temperature (°C).

	**Spring**			**Fall**		
	Biomass	Relative *F*	Temperature	Biomass	Relative *F*	Temperature
XCG	-3.25	-4.36	-0.18	-1.01	-3.74	-6.10
YCG	-1.24	-1.69	-0.05	8.64	1.26	-0.90
Inertia	-440.26	-42.34	-10.61	40.03	137.32	-304.24
Depth	-6.42	-2.89	-2.18	15.65	-2.08	1.45
PA	699.35	-6813.92	5170.80	10950.10	-3336.08	3620.09

Species: Red Hake (*Urophycis chuss*)
Stock: Southern

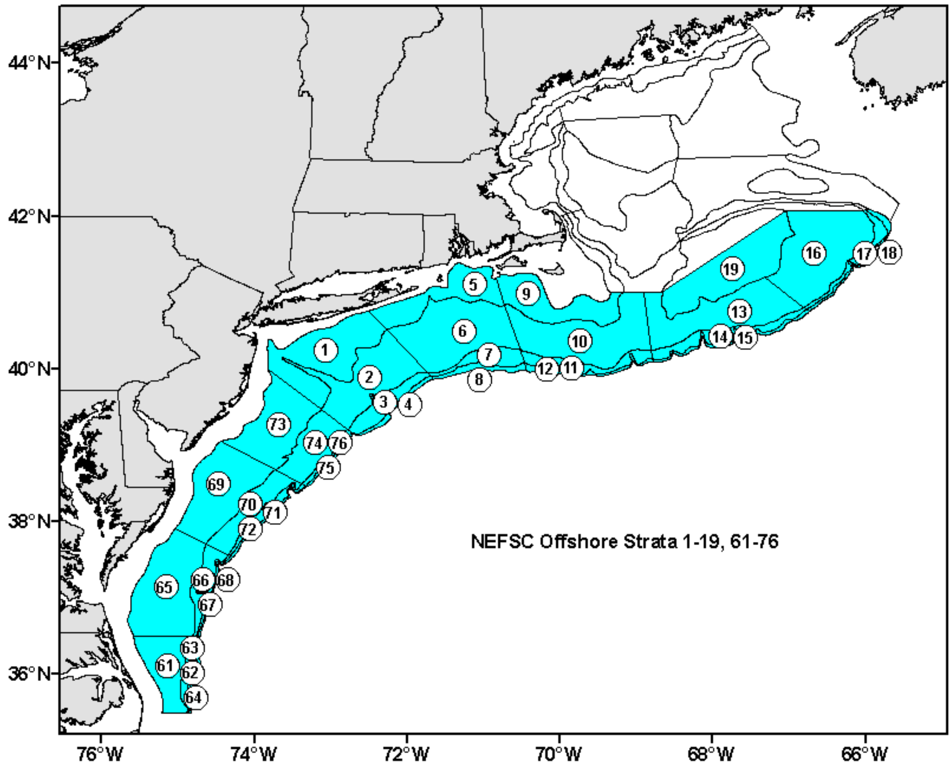

Northeast Fisheries Science Center bottom trawl survey offshore strata on the continental shelf of the Northeast United States. Red hake (southern) stock strata are shown in blue.

NEFSC surveys used in the most recent assessment: Spring only

Link to most recent benchmark assessment:

Northeast Fisheries Science Center. 51st Northeast regional stock assessment workshop (51st SAW) *assessment report*. US Dept Commer, Northeast Fish Sci Cent Ref Doc. 2011; 11-02. 856 pp. Available from: http://www.nefsc.noaa.gov/publications/crd/crd1102/.

Link to most recent assessment update:

New England Fisheries Management Council. Small-mesh multispecies fishing year 2016-2017 specifications supplemental information report (SIR), and regulatory flexibility analysis (RFA). 2016. 33 pp. Available from: http://s3.amazonaws.com/nefmc.org/2016-2017-Specifications-Supplemental-Information-Report.pdf.

Time-series analysis

Estimate, standard error, *t*-value and *p*-value for linear regressions of red hake (southern) center of gravity (XCG, YCG), inertia, depth and positive area (PA) as a function of year. *P*-values < 0.05 are in bold. Corresponding time series plots are shown below, with significant linear fits indicated by a solid line. Sample sizes: spring (n = 36); fall (n = 40).

	Spring				Fall			
	Estimate	SE	*t*-value	*p*-value	Estimate	SE	*t*-value	*p*-value
XCG	6.18	0.97	6.36	**< 0.001**	2.68	0.79	3.38	**0.002**
YCG	1.76	0.30	5.95	**< 0.001**	0.28	0.21	1.35	0.185
Inertia	-107.47	93.65	-1.15	0.259	190.47	129.04	1.48	0.148
Depth	1.03	0.36	2.84	**0.008**	0.51	0.16	3.16	**0.003**
PA	53.54	163.78	0.33	0.746	138.09	112.54	1.23	0.227

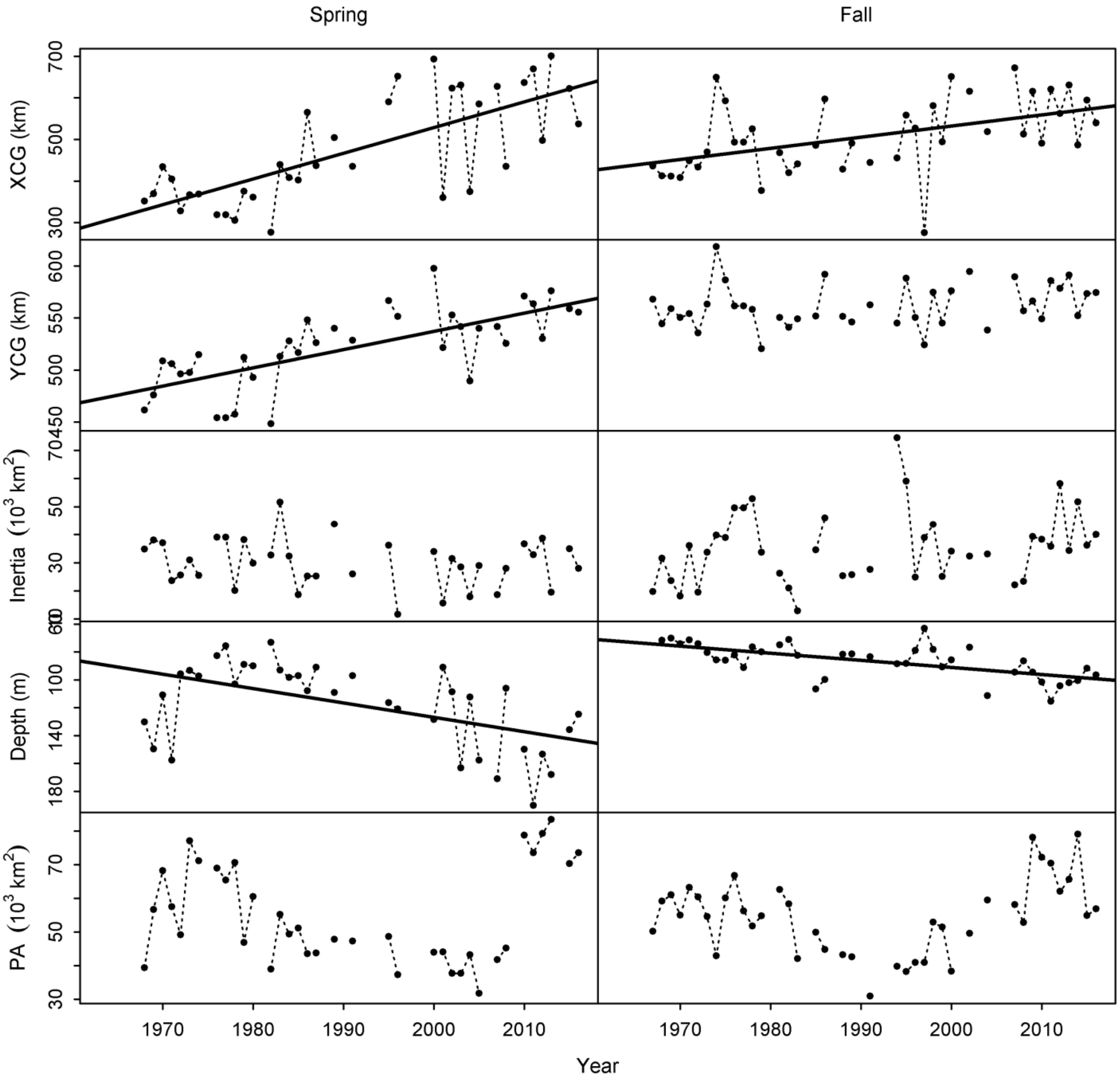

Predictor variables

Time-series of predictor variables for red hake (southern). Predictors and associated units are: Northeast Fisheries Science Center (NEFSC) bottom trawl survey stratified mean kg per tow (biomass), catch/NEFSC stratified mean kg per tow (relative *F*) and NEFSC stratified mean bottom temperature (°C). Data for all three predictors were set to NA for years in which there were insufficient bottom temperature recordings to calculate a stratified mean. Sample sizes: spring (n = 20); fall (n = 21).

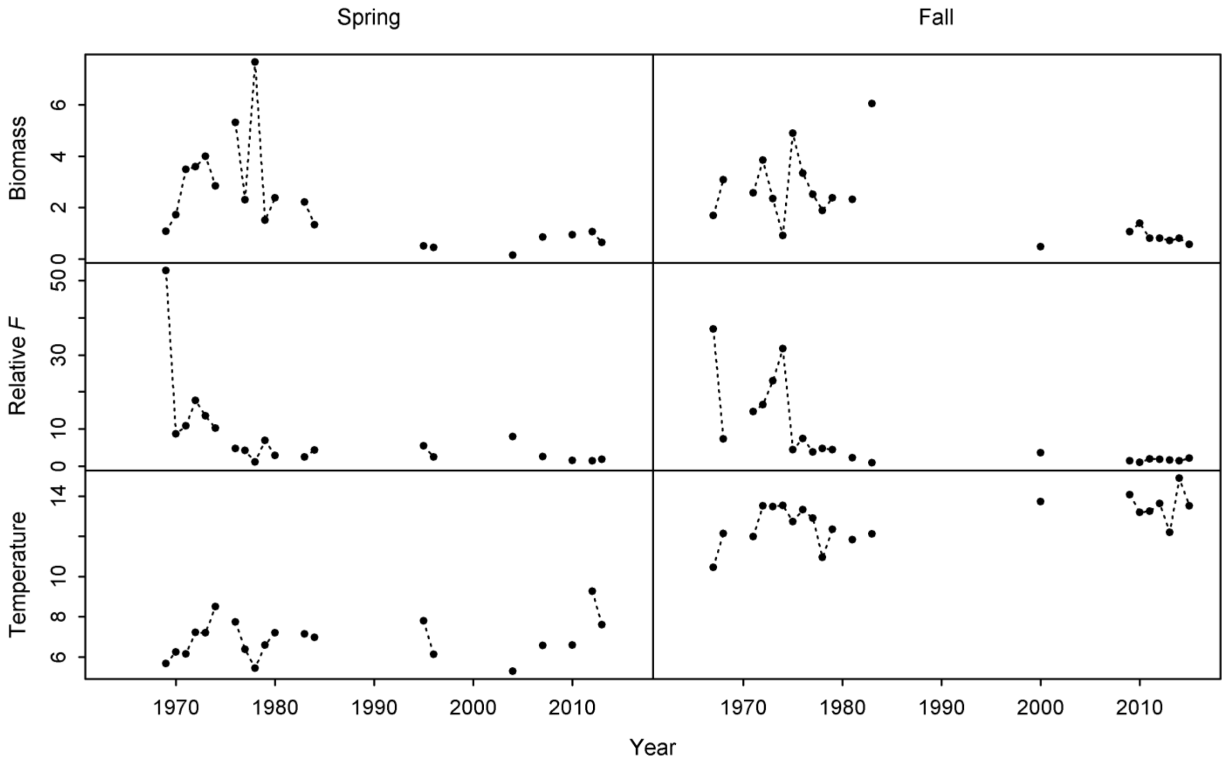

Relative importance of predictors on spatial distribution

Relative importance of predictor variables on spatial indicators for red hake (southern). Spatial indicators and associated units are: geographically referenced longitude and latitude of the center of gravity (XCG and YCG, respectively; km), inertia (km^2), depth (m) and positive area (PA; km^2). Predictor variables are: Northeast Fisheries Science Center (NEFSC) bottom trawl survey stratified mean kg per tow (biomass; kg), catch/NEFSC stratified mean kg per tow (relative *F*) and NEFSC stratified mean bottom temperature (°C). Predictor variables with the highest summed Akaike weights for each indicator are in bold.

	**Spring**			**Fall**		
Predictor	Biomass	Relative *F*	Temperature	Biomass	Relative *F*	Temperature
XCG	0.40	**0.42**	0.18	**0.98**	0.16	0.16
YCG	**0.55**	0.25	0.22	**0.94**	0.19	0.42
Inertia	**0.29**	0.17	0.25	0.31	0.16	**0.45**
Depth	0.17	0.17	**0.24**	0.31	**0.35**	0.33
PA	**0.45**	**0.45**	0.18	0.26	0.23	**0.31**

Model-averaged parameter estimates

Model-averaged predictor estimates by spatial indicator for red hake (southern). Spatial indicators and associated units are: geographically referenced longitude and latitude of the center of gravity (XCG and YCG, respectively; km), inertia (km^2), depth (m) and positive area (PA; km^2). Predictor variables are: Northeast Fisheries Science Center (NEFSC) bottom trawl survey stratified mean kg per tow (biomass; kg), catch/NEFSC stratified mean kg per tow (relative *F*) and NEFSC stratified mean bottom temperature (°C).

	**Spring**			**Fall**		
	Biomass	Relative *F*	Temperature	Biomass	Relative *F*	Temperature
XCG	-22.75	-19.22	-1.85	-79.93	-1.36	1.13
YCG	-9.42	-2.11	1.49	-19.28	0.64	-2.88
Inertia	774.86	122.14	716.58	-1487.41	-114.00	1774.50
Depth	0.74	-0.36	-1.36	-1.35	-1.47	0.77
PA	2944.08	-2742.04	456.29	1239.30	-703.24	952.64

Species: Silver Hake (*Merluccius bilinearis*)
Stock: Northern

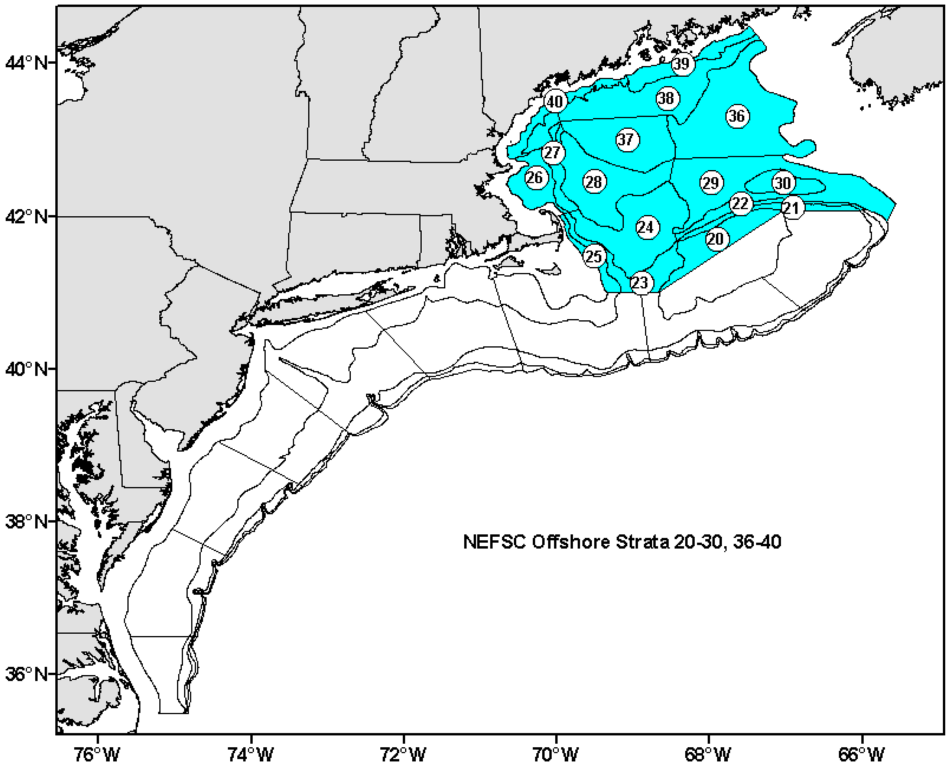

Northeast Fisheries Science Center bottom trawl survey offshore strata on the continental shelf of the Northeast United States. Silver hake (northern) stock strata are shown in blue.

NEFSC surveys used in the most recent assessment: Fall only

Link to most recent benchmark assessment:
Northeast Fisheries Science Center. 51st Northeast regional stock assessment workshop (51st SAW) *assessment report*. US Dept Commer, Northeast Fish Sci Cent Ref Doc. 2011; 11-02. 856 pp. Available from: http://www.nefsc.noaa.gov/publications/crd/crd1102/.

Link to most recent assessment update:
New England Fisheries Management Council. Small-mesh multispecies fishing year 2015-2017 specifications environmental assessment regulatory impact review and initial regulatory flexibility analysis. 2015. 136 pp. Available from: http://s3.amazonaws.com/nefmc.org/2015-2017-Specificatins-Document-2.pdf.

Time-series analysis

Estimate, standard error, *t*-value and *p*-value for linear regressions of silver hake (northern) center of gravity (XCG, YCG), inertia, depth and positive area (PA) as a function of year. *P*-values < 0.05 are in bold. Corresponding time series plots are shown below, with significant linear fits indicated by a solid line. Sample sizes: spring (n = 48); fall (n = 54).

	Spring				Fall			
	Estimate	SE	*t*-value	*p*-value	Estimate	SE	*t*-value	*p*-value
XCG	0.15	0.31	0.47	0.643	-0.01	0.18	-0.07	0.944
YCG	0.72	0.24	2.97	**0.005**	0.76	0.25	3.05	**0.004**
Inertia	42.33	19.71	2.15	**0.037**	1.06	15.40	0.07	0.945
Depth	-0.63	0.28	-2.25	**0.029**	-0.11	0.17	-0.61	0.547
PA	606.28	168.75	3.59	**< 0.001**	205.82	45.58	4.52	**< 0.001**

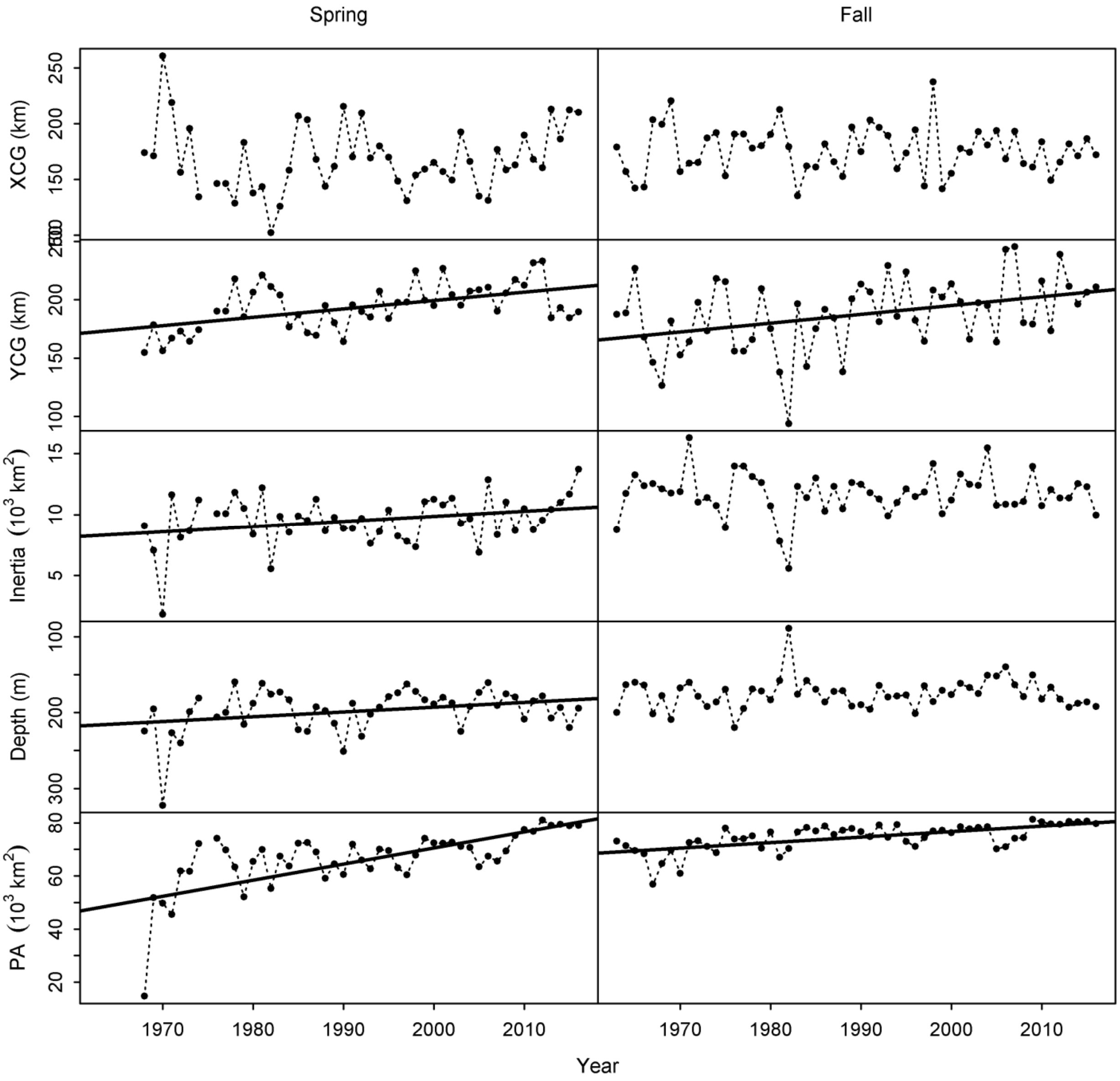

Predictor variables

Time-series of predictor variables for silver hake (northern). Predictors and associated units are: Northeast Fisheries Science Center (NEFSC) bottom trawl survey stratified mean kg per tow (biomass), catch/NEFSC stratified mean kg per tow (relative *F*) and NEFSC stratified mean bottom temperature (°C). Data for all three predictors were set to NA for years in which there were insufficient bottom temperature recordings to calculate a stratified mean. Sample sizes: spring (*n* = 41); fall (*n* = 46).

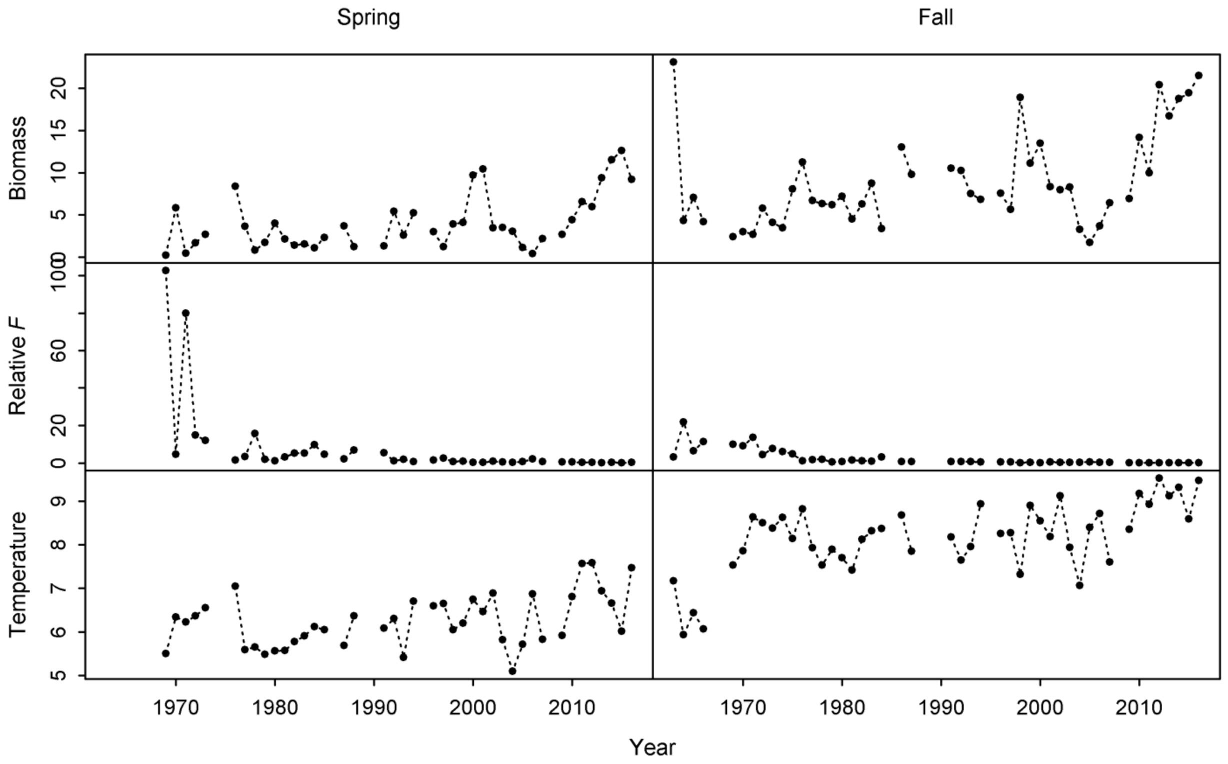

Relative importance of predictors on spatial distribution

Relative importance of predictor variables on spatial indicators for silver hake (northern). Spatial indicators and associated units are: geographically referenced longitude and latitude of the center of gravity (XCG and YCG, respectively; km), inertia (km^2), depth (m) and positive area (PA; km^2). Predictor variables are: Northeast Fisheries Science Center (NEFSC) bottom trawl survey stratified mean kg per tow (biomass; kg), catch/NEFSC stratified mean kg per tow (relative *F*) and NEFSC stratified mean bottom temperature (°C). Predictor variables with the highest summed Akaike weights for each indicator are in bold.

	**Spring**			**Fall**		
Predictor	Biomass	Relative *F*	Temperature	Biomass	Relative *F*	Temperature
XCG	**0.95**	0.39	0.23	0.25	**0.39**	0.27
YCG	0.78	**0.80**	0.31	**0.66**	0.52	0.24
Inertia	0.29	**0.58**	0.28	**0.30**	0.26	**0.30**
Depth	**0.97**	0.50	0.27	**0.95**	0.60	0.35
PA	0.27	**0.96**	0.55	0.94	**0.97**	0.43

Model-averaged parameter estimates

Model-averaged predictor estimates by spatial indicator for silver hake (northern). Spatial indicators and associated units are: geographically referenced longitude and latitude of the center of gravity (XCG and YCG, respectively; km), inertia (km^2), depth (m) and positive area (PA; km^2). Predictor variables are: Northeast Fisheries Science Center (NEFSC) bottom trawl survey stratified mean kg per tow (biomass; kg), catch/NEFSC stratified mean kg per tow (relative *F*) and NEFSC stratified mean bottom temperature (°C).

	**Spring**			**Fall**		
	Biomass	Relative *F*	Temperature	Biomass	Relative *F*	Temperature
XCG	17.86	2.75	-0.68	-0.50	-1.10	-0.78
YCG	-9.40	-8.18	1.39	11.07	-3.42	-0.61
Inertia	111.63	-250.52	131.91	-129.87	-33.77	-108.94
Depth	24.91	5.47	-1.34	15.88	3.02	1.63
PA	392.62	-3593.93	1457.86	2555.15	-1311.50	398.52

Species: Silver Hake (*Merluccius bilinearis*)
Stock: Southern

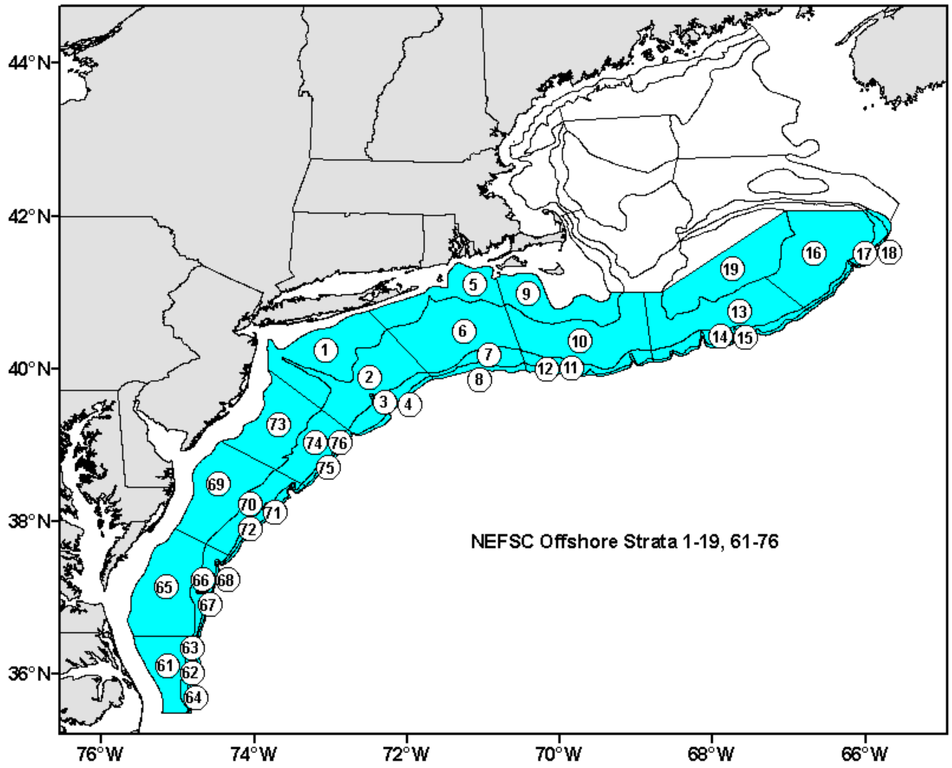

Northeast Fisheries Science Center bottom trawl survey offshore strata on the continental shelf of the Northeast United States. Silver hake (southern) stock strata are shown in blue.

NEFSC surveys used in the most recent assessment: Fall only

Link to most recent benchmark assessment:
Northeast Fisheries Science Center. 51st Northeast regional stock assessment workshop (51st SAW) *assessment report*. US Dept Commer, Northeast Fish Sci Cent Ref Doc. 2011; 11-02. 856 pp. Available from: http://www.nefsc.noaa.gov/publications/crd/crd1102/.

Link to most recent assessment update:
New England Fisheries Management Council. Small-mesh multispecies fishing year 2015-2017 specifications environmental assessment regulatory impact review and initial regulatory flexibility analysis. 2015. 136 pp. Available from: http://s3.amazonaws.com/nefmc.org/2015-2017-Specificatins-Document-2.pdf

Time-series analysis

Slope (β), standard error, *t*-value and *p*-value for linear regressions of silver hake (southern) center of gravity (XCG, YCG), inertia, depth and positive area (PA) as a function of year. *P*-values < 0.05 are in bold. Corresponding time series plots are shown below, with significant linear fits indicated by a solid line. Sample sizes: spring (n = 37); fall (n = 40).

	Spring				Fall			
	Estimate	SE	*t*-value	*p*-value	Estimate	SE	*t*-value	*p*-value
XCG	5.56	1.60	3.48	**0.001**	2.90	0.60	4.86	**< 0.001**
YCG	2.37	0.59	4.04	**< 0.001**	0.84	0.17	4.81	**< 0.001**
Inertia	-198.63	143.79	-1.38	0.176	67.61	115.46	0.59	0.562
Depth	0.29	0.28	1.03	0.309	0.20	0.11	1.76	0.087
PA	80.23	171.23	0.47	0.642	-12.20	105.48	-0.12	0.909

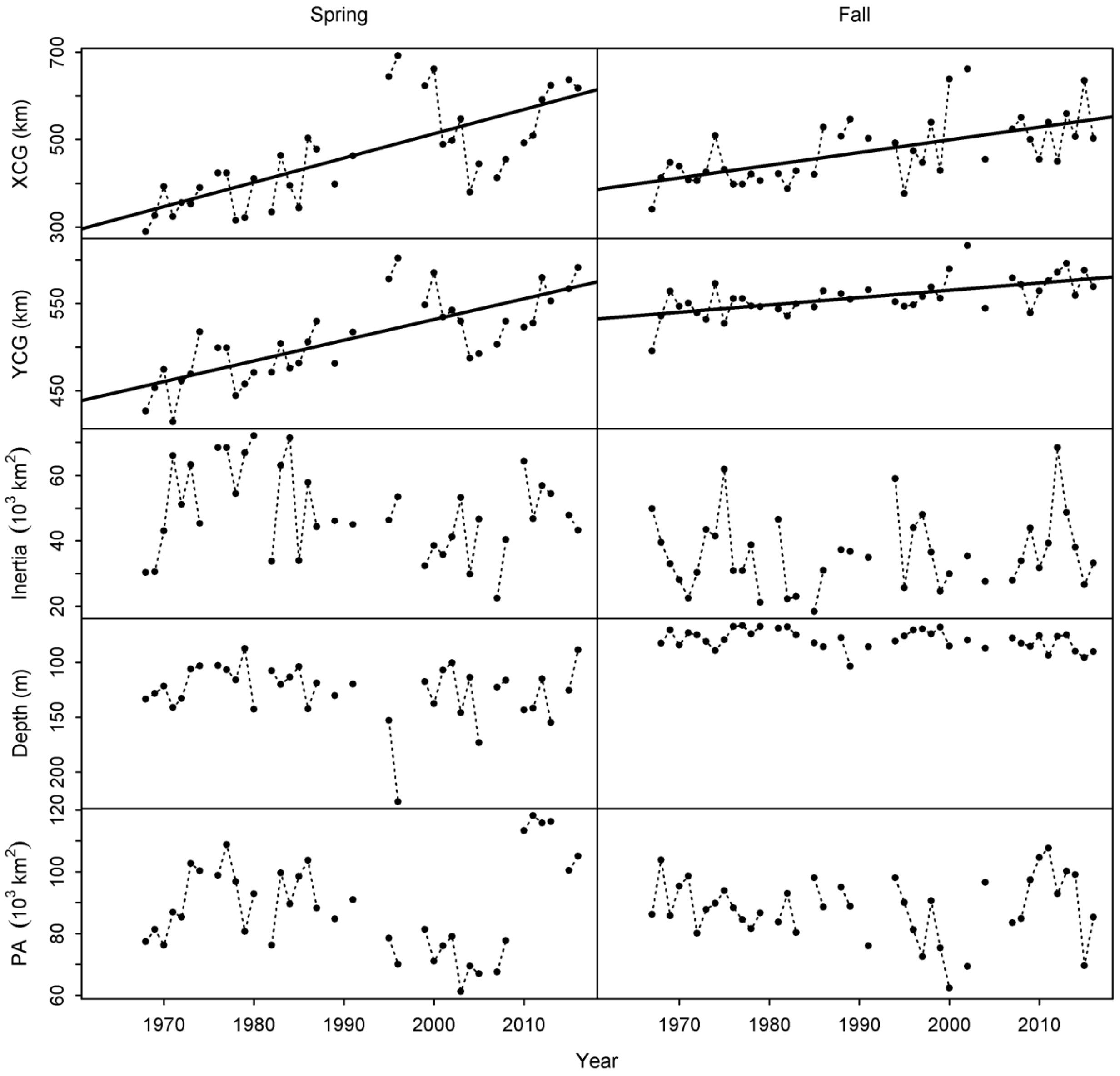

Predictor variables

Time-series of predictor variables for silver hake (southern). Predictors and associated units are: Northeast Fisheries Science Center (NEFSC) bottom trawl survey stratified mean kg per tow (biomass), catch/NEFSC stratified mean kg per tow (relative *F*) and NEFSC stratified mean bottom temperature (°C). Data for all three predictors were set to NA for years in which there were insufficient bottom temperature recordings to calculate a stratified mean. Sample sizes: spring (*n* = 20); fall (*n* = 21).

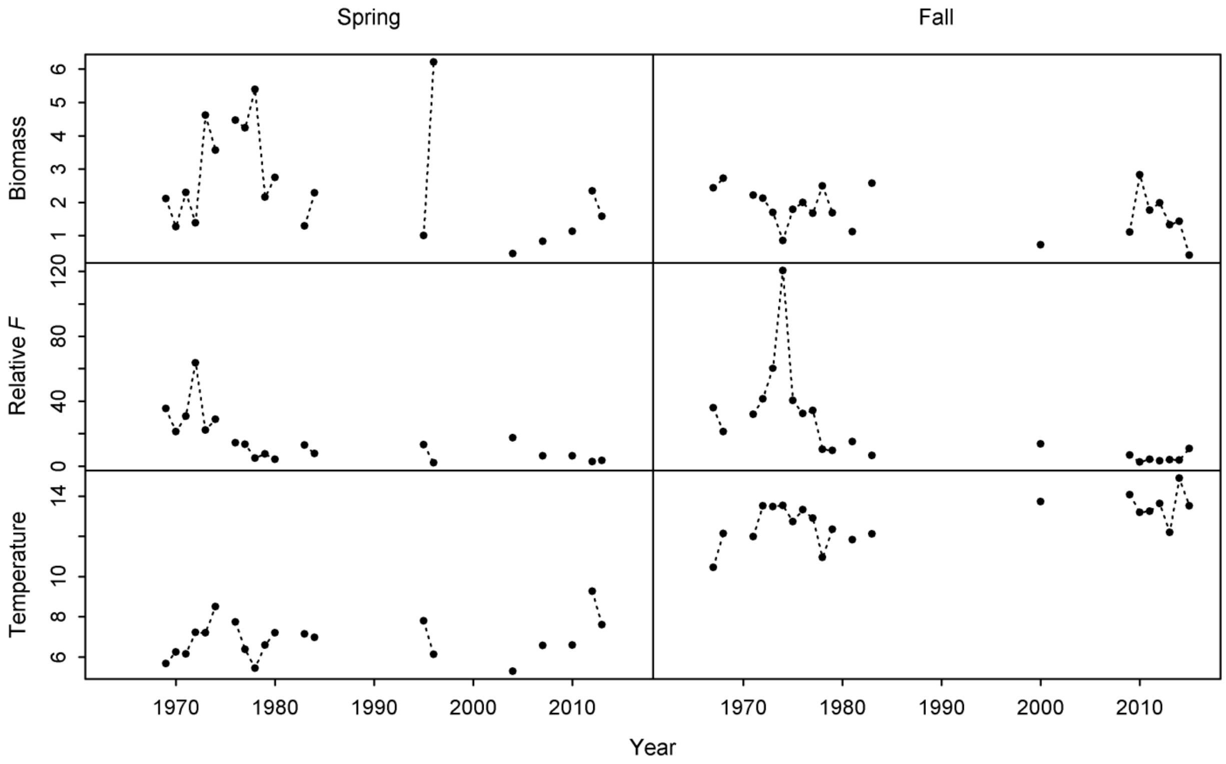

Relative importance of predictors on spatial distribution

Relative importance of predictor variables on spatial indicators for silver hake (southern). Spatial indicators and associated units are: geographically referenced longitude and latitude of the center of gravity (XCG and YCG, respectively; km), inertia (km^2), depth (m) and positive area (PA; km^2). Predictor variables are: Northeast Fisheries Science Center (NEFSC) bottom trawl survey stratified mean kg per tow (biomass; kg), catch/NEFSC stratified mean kg per tow (relative *F*) and NEFSC stratified mean bottom temperature (°C). Predictor variables with the highest summed Akaike weights for each indicator are in bold.

	**Spring**			**Fall**		
Predictor	Biomass	Relative *F*	Temperature	Biomass	Relative *F*	Temperature
XCG	0.18	0.33	**0.43**	**1.00**	0.83	0.46
YCG	0.16	0.30	**0.85**	**0.86**	0.71	0.14
Inertia	**0.60**	0.19	0.19	0.16	**0.17**	**0.17**
Depth	0.25	**0.61**	0.36	**0.62**	0.16	0.54
PA	**0.26**	0.17	0.18	**0.75**	0.16	0.15

Model-averaged parameter estimates

Model-averaged predictor estimates by spatial indicator for silver hake (southern). Spatial indicators and associated units are: geographically referenced longitude and latitude of the center of gravity (XCG and YCG, respectively; km), inertia (km^2), depth (m) and positive area (PA; km^2). Predictor variables are: Northeast Fisheries Science Center (NEFSC) bottom trawl survey stratified mean kg per tow (biomass; kg), catch/NEFSC stratified mean kg per tow (relative *F*) and NEFSC stratified mean bottom temperature (°C).

	**Spring**			**Fall**		
	Biomass	Relative *F*	Temperature	Biomass	Relative *F*	Temperature
XCG	-1.00	-10.57	10.75	-114.72	-23.29	3.74
YCG	-0.17	-4.44	16.36	-18.66	-6.96	0.14
Inertia	5834.55	-539.81	440.29	221.68	-179.62	185.05
Depth	3.06	-9.97	-3.35	-4.89	-0.14	2.72
PA	1273.35	232.26	350.86	8621.01	-246.27	60.81

Species: White Hake (*Urophycis tenuis*)
Stock: Unit

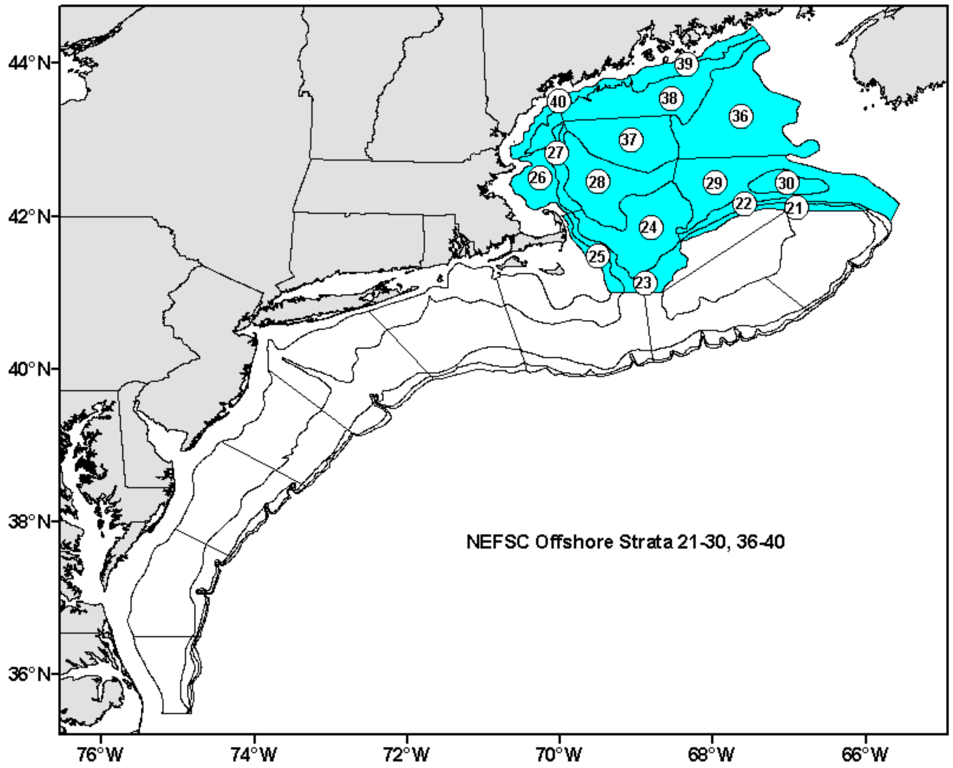

Northeast Fisheries Science Center bottom trawl survey offshore strata on the continental shelf of the Northeast United States. White hake (unit) stock strata are shown in blue.

NEFSC surveys used in the most recent assessment: Spring & Fall

<u>Link to most recent benchmark assessment</u>:

Northeast Fisheries Science Center. 56th Northeast regional stock assessment workshop (56th SAW) *assessment report*. US Dept Commer, Northeast Fish Sci Cent Ref Doc. 2013; 13-10. 868 pp. Available from: http://nefsc.noaa.gov/publications/crd/crd1310/.

<u>Link to most recent assessment update</u>:

Northeast Fisheries Science Center. Operational assessment of 19 Northeast groundfish stocks, updated through 2016. US Dept Commer, Northeast Fish Sci Cent Ref Doc. 2017; 17-17. 259 pp. Available from: http://www.nefsc.noaa.gov/publications/crd/crd1717/.

Time-series analysis

Estimate, standard error, *t*-value and *p*-value for linear regressions of white hake (unit) center of gravity (XCG, YCG), inertia, depth and positive area (PA) as a function of year. *P*-values < 0.05 are in bold. Corresponding time series plots are shown below, with significant linear fits indicated by a solid line. Sample sizes: spring (*n* = 48); fall (*n* = 54).

	**Spring**				**Fall**			
	Estimate	SE	*t*-value	*p*-value	Estimate	SE	*t*-value	*p*-value
XCG	0.73	0.29	2.48	**0.017**	0.53	0.18	2.96	**0.005**
YCG	-0.11	0.16	-0.73	0.467	-0.17	0.10	-1.71	0.093
Inertia	-13.76	16.80	-0.82	0.417	-33.67	17.18	-1.96	0.056
Depth	0.45	0.23	1.99	0.053	0.51	0.09	5.38	**< 0.001**
PA	57.49	85.81	0.67	0.506	171.68	94.52	1.82	0.075

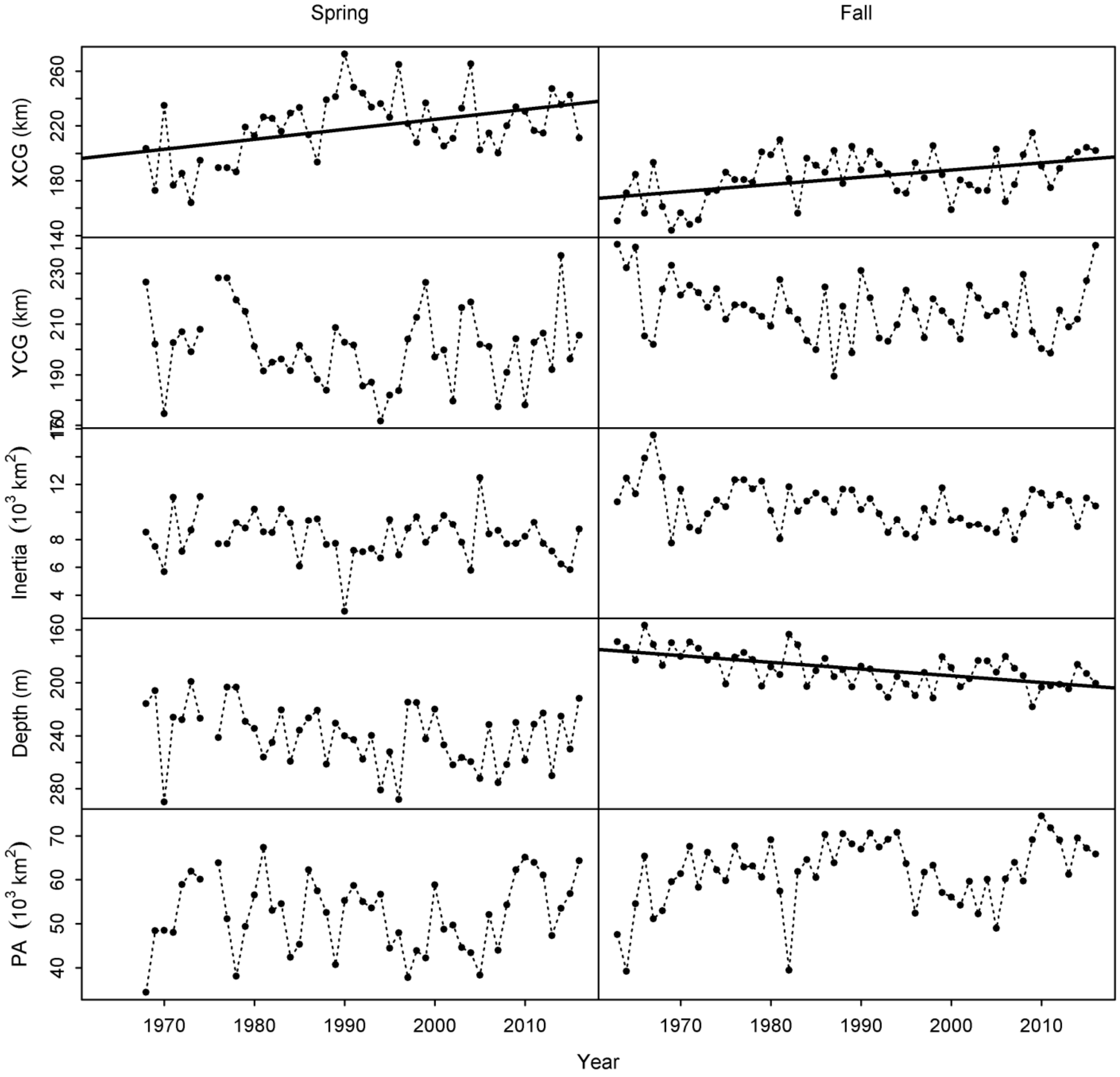

Predictor variables

Time-series of predictor variables for white hake (unit). Predictors and associated units are: Northeast Fisheries Science Center (NEFSC) bottom trawl survey stratified mean kg per tow (biomass), catch/NEFSC stratified mean kg per tow (relative *F*) and NEFSC stratified mean bottom temperature (°C). Data for all three predictors were set to NA for years in which there were insufficient bottom temperature recordings to calculate a stratified mean. Sample sizes: spring (*n* = 41); fall (*n* = 46).

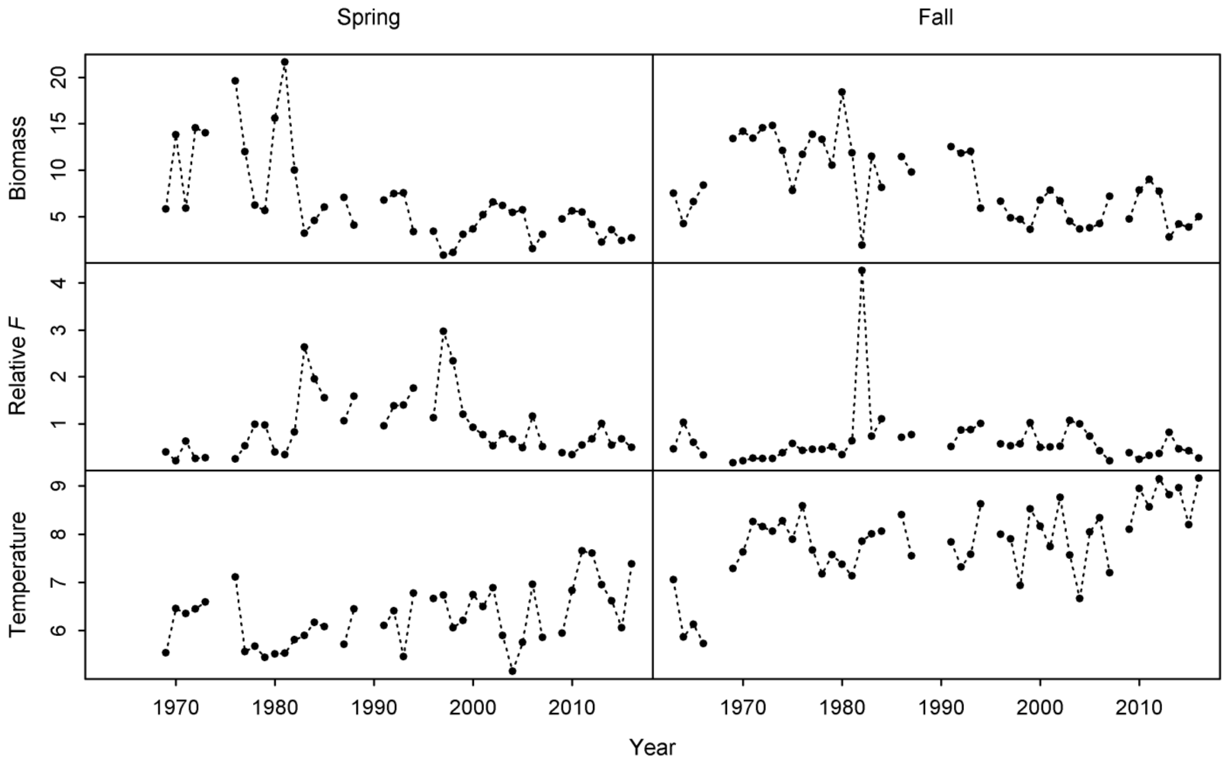

Relative importance of predictors on spatial distribution

Relative importance of predictor variables on spatial indicators for white hake (unit). Spatial indicators and associated units are: geographically referenced longitude and latitude of the center of gravity (XCG and YCG, respectively; km), inertia (km^2), depth (m) and positive area (PA; km^2). Predictor variables are: Northeast Fisheries Science Center (NEFSC) bottom trawl survey stratified mean kg per tow (biomass; kg), catch/NEFSC stratified mean kg per tow (relative *F*) and NEFSC stratified mean bottom temperature (°C). Predictor variables with the highest summed Akaike weights for each indicator are in bold.

	**Spring**			**Fall**		
Predictor	Biomass	Relative *F*	Temperature	Biomass	Relative *F*	Temperature
XCG	0.25	**0.43**	0.22	0.34	**0.38**	0.22
YCG	0.22	0.23	**0.28**	0.24	**0.33**	0.26
Inertia	0.22	**0.24**	**0.24**	**0.37**	**0.37**	0.23
Depth	**0.22**	**0.22**	**0.22**	0.23	0.23	**0.25**
PA	0.66	0.50	**0.91**	0.52	**0.82**	0.43

Model-averaged parameter estimates

Model-averaged predictor estimates by spatial indicator for white hake (unit). Spatial indicators and associated units are: geographically referenced longitude and latitude of the center of gravity (XCG and YCG, respectively; km), inertia (km^2), depth (m) and positive area (PA; km^2). Predictor variables are: Northeast Fisheries Science Center (NEFSC) bottom trawl survey stratified mean kg per tow (biomass; kg), catch/NEFSC stratified mean kg per tow (relative *F*) and NEFSC stratified mean bottom temperature (°C).

	**Spring**			**Fall**		
	Biomass	Relative *F*	Temperature	Biomass	Relative *F*	Temperature
XCG	-1.06	4.74	-0.01	-1.97	2.38	0.01
YCG	-0.07	-0.31	-0.92	-0.20	-1.20	0.57
Inertia	-8.67	40.12	-40.19	-241.23	208.56	-7.42
Depth	0.05	0.07	0.17	-0.09	-0.08	0.68
PA	3163.54	-2071.75	4735.55	3011.93	-6049.66	874.97

Species: Yellowtail Flounder (*Limanda ferruginea*)
Stock: Georges Bank

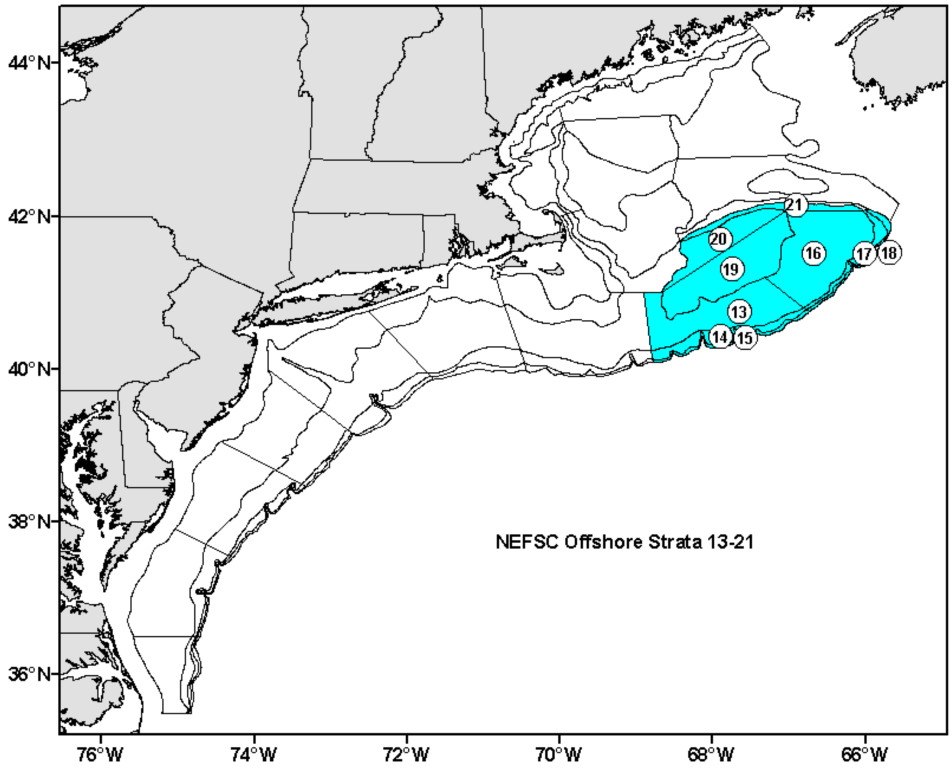

Northeast Fisheries Science Center bottom trawl survey offshore strata on the continental shelf of the Northeast United States. Yellowtail flounder (Georges Bank) stock strata are shown in blue.

NEFSC surveys used in the most recent assessment: Spring & Fall

Link to most recent benchmark assessment:
Legault CM, Alade L, Gross WE, Stone HH. Stock assessment of Georges Bank yellowtail flounder for 2014. Transboundary Resources Assessment Committee Ref Doc. 2014; 2014/01. 214 pp. Available from: http://www.nefsc.noaa.gov/saw/trac/TRD_2014_01_E_.pdf.

Link to most recent assessment update:
Legault CM, McCurdy QM. Stock Assessment of Georges Bank Yellowtail Flounder for 2017. Transboundary Resources Assessment Committee Work Pap. 2017; 2017/03. 66 pp. Available from: https://www.nefsc.noaa.gov/saw/trac/wp3_legault_gb_yellowtail_assessment.pdf.

Time-series analysis

Estimate, standard error, *t*-value and *p*-value for linear regressions of yellowtail flounder (Georges Bank) center of gravity (XCG, YCG), inertia, depth and positive area (PA) as a function of year. *P*-values < 0.05 are in bold. Corresponding time series plots are shown below, with significant linear fits indicated by a solid line. Sample sizes: spring (n = 47); fall (n = 54).

	**Spring**				**Fall**			
	Estimate	SE	*t*-value	*p*-value	Estimate	SE	*t*-value	*p*-value
XCG	0.94	0.37	2.54	**0.015**	1.21	0.25	4.78	**< 0.001**
YCG	0.54	0.29	1.89	0.065	0.63	0.24	2.65	**0.011**
Inertia	-57.08	18.27	-3.12	**0.003**	-63.59	14.51	-4.38	**< 0.001**
Depth	0.24	0.08	2.89	**0.006**	0.18	0.09	1.93	0.060
PA	-38.74	45.62	-0.85	0.400	-208.55	101.56	-2.05	**0.045**

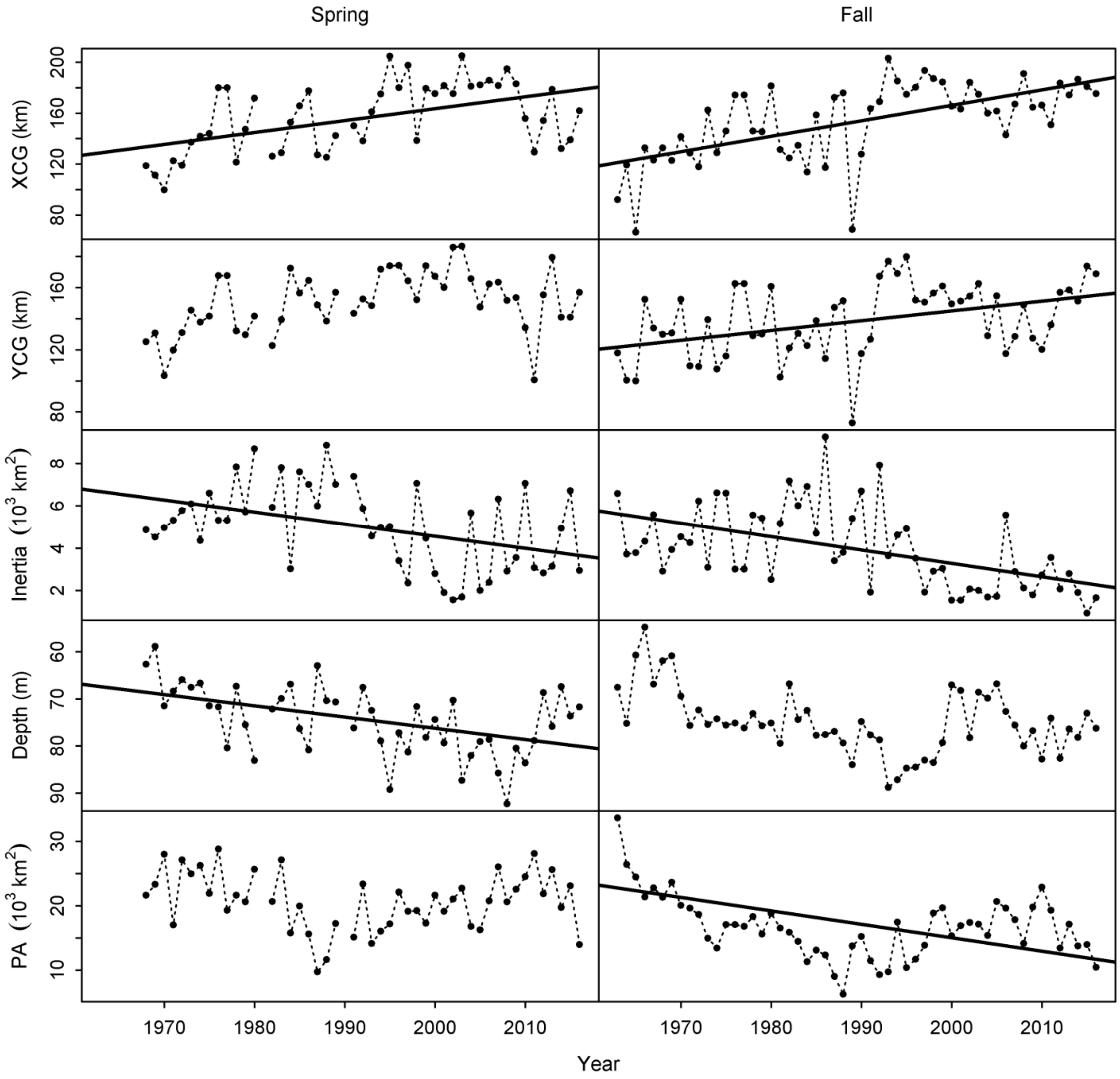

Predictor variables

Time-series of predictor variables for yellowtail flounder (Georges Bank). Predictors and associated units are: Northeast Fisheries Science Center (NEFSC) bottom trawl survey stratified mean kg per tow (biomass), catch/NEFSC stratified mean kg per tow (relative *F*) and NEFSC stratified mean bottom temperature (°C). Data for all three predictors were set to NA for years in which there were insufficient bottom temperature recordings to calculate a stratified mean. Sample sizes: spring (n = 37); fall (n = 41).

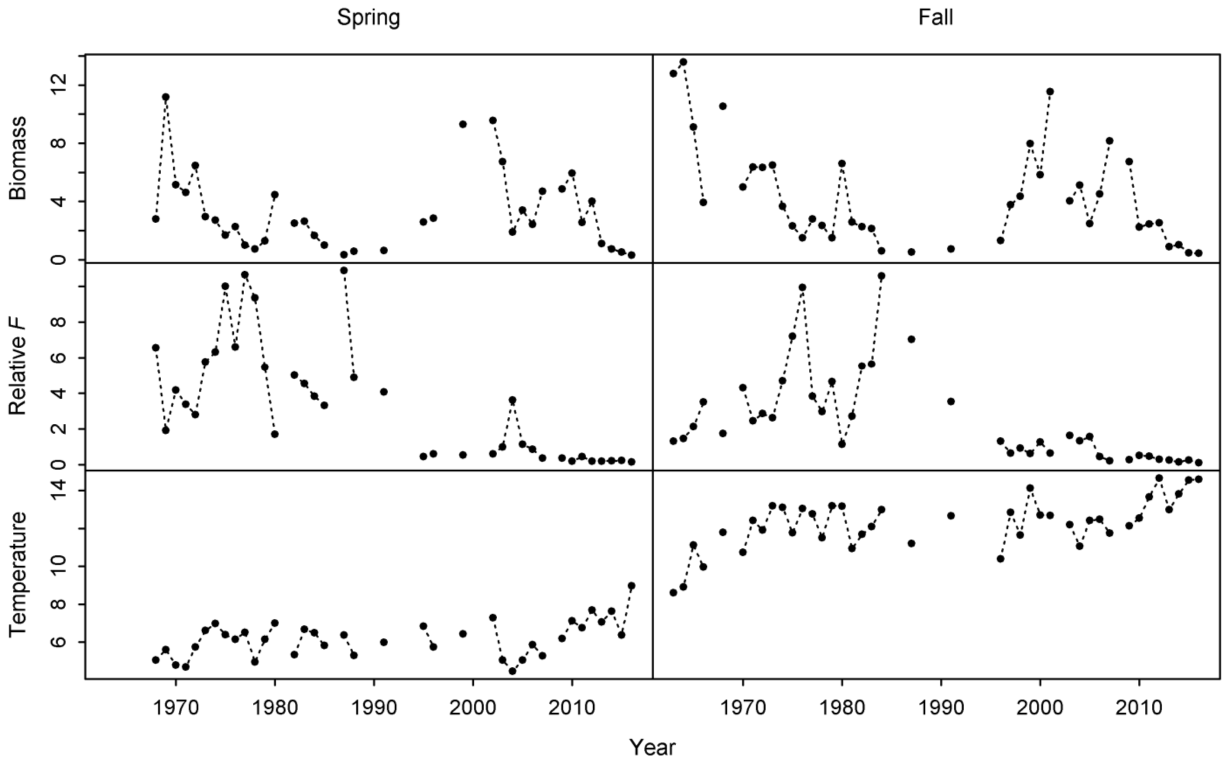

Relative importance of predictors on spatial distribution

Relative importance of predictor variables on spatial indicators for yellowtail flounder (Georges Bank). Spatial indicators and associated units are: geographically referenced longitude and latitude of the center of gravity (XCG and YCG, respectively; km), inertia (km^2), depth (m) and positive area (PA; km^2). Predictor variables are: Northeast Fisheries Science Center (NEFSC) bottom trawl survey stratified mean kg per tow (biomass; kg), catch/NEFSC stratified mean kg per tow (relative *F*) and NEFSC stratified mean bottom temperature (°C). Predictor variables with the highest summed Akaike weights for each indicator are in bold.

	**Spring**			**Fall**		
Predictor	Biomass	Relative *F*	Temperature	Biomass	Relative *F*	Temperature
XCG	0.23	**0.79**	0.27	0.41	**0.70**	0.48
YCG	0.25	**0.57**	0.46	0.42	0.40	**0.77**
Inertia	0.69	**0.92**	0.23	0.22	**0.94**	0.48
Depth	0.22	**0.67**	0.42	0.29	**0.56**	0.22
PA	**0.97**	0.23	0.32	**0.70**	0.25	0.66

Model-averaged parameter estimates

Model-averaged predictor estimates by spatial indicator for yellowtail flounder (Georges Bank). Spatial indicators and associated units are: geographically referenced longitude and latitude of the center of gravity (XCG and YCG, respectively; km), inertia (km^2), depth (m) and positive area (PA; km^2). Predictor variables are: Northeast Fisheries Science Center (NEFSC) bottom trawl survey stratified mean kg per tow (biomass; kg), catch/NEFSC stratified mean kg per tow (relative *F*) and NEFSC stratified mean bottom temperature (°C).

	**Spring**			**Fall**		
	Biomass	Relative *F*	Temperature	Biomass	Relative *F*	Temperature
XCG	0.54	-8.10	0.83	-2.70	-6.23	2.77
YCG	0.67	-3.71	2.49	-2.13	-1.59	4.53
Inertia	-421.77	537.74	-1.25	9.35	742.26	-145.84
Depth	0.07	-1.58	-0.76	-0.33	-1.12	0.02
PA	2507.68	26.89	375.32	1796.86	-38.24	-605.08

Species: Yellowtail Flounder (*Limanda ferruginea*)
Stock: Southern New England-Mid Atlantic

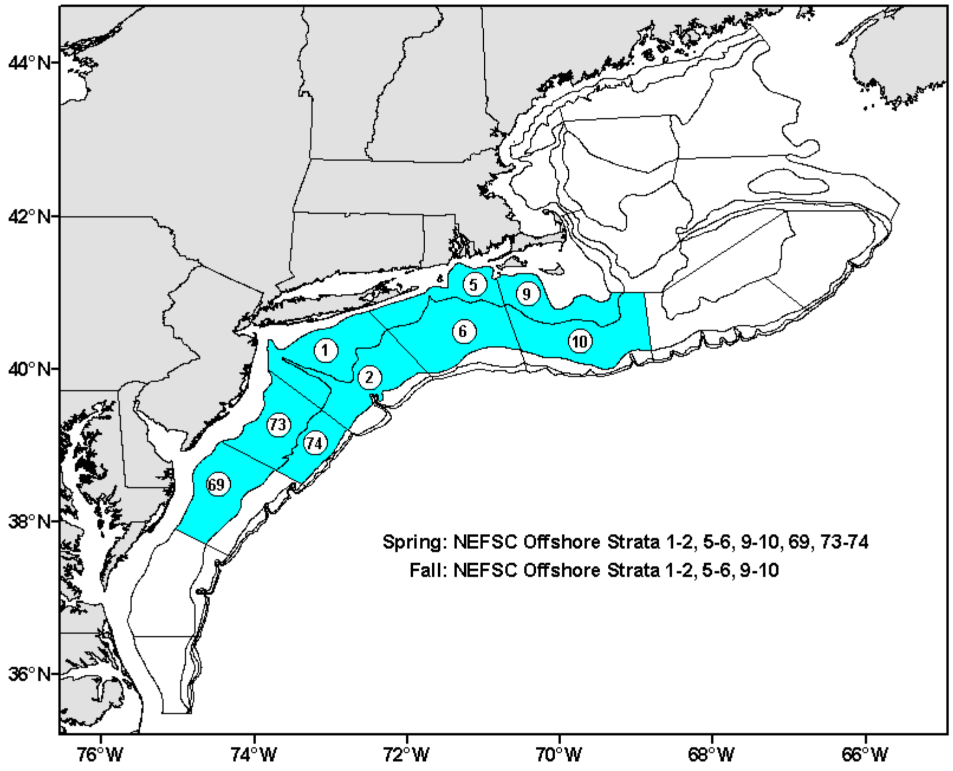

Northeast Fisheries Science Center bottom trawl survey offshore strata on the continental shelf of the Northeast United States. Yellowtail flounder (Southern New England-Mid Atlantic) stock strata are shown in blue.

NEFSC surveys used in the most recent assessment: Spring & Fall

<u>Link to most recent benchmark assessment</u>:

Northeast Fisheries Science Center. 54th Northeast regional stock assessment workshop (54th SAW) *assessment report*. US Dept Commer, Northeast Fish Sci Cent Ref Doc. 2012; 12-18. 600 pp. Available from: http://www.nefsc.noaa.gov/publications/crd/crd1218/.

<u>Link to most recent assessment update</u>:

Northeast Fisheries Science Center. Operational assessment of 19 Northeast groundfish stocks, updated through 2016. US Dept Commer, Northeast Fish Sci Cent Ref Doc. 2017; 17-17. 259 pp. Available from: http://www.nefsc.noaa.gov/publications/crd/crd1717/.

Time-series analysis

Estimate, standard error, *t*-value and *p*-value for linear regressions of yellowtail flounder (Southern New England-Mid Atlantic) center of gravity (XCG, YCG), inertia, depth and positive area (PA) as a function of year. *P*-values < 0.05 are in bold. Corresponding time series plots are shown below, with significant linear fits indicated by a solid line. Sample sizes: spring (*n* = 49); fall (*n* = 54).

	**Spring**				**Fall**			
	Estimate	SE	*t*-value	*p*-value	Estimate	SE	*t*-value	*p*-value
XCG	1.16	0.37	3.10	**0.003**	0.26	0.46	0.57	0.570
YCG	0.85	0.17	4.95	**< 0.001**	0.28	0.15	1.85	0.070
Inertia	-193.15	37.49	-5.15	**< 0.001**	-119.23	36.32	-3.28	**0.002**
Depth	0.08	0.04	2.25	**0.029**	0.10	0.04	2.38	**0.021**
PA	-664.93	196.87	-3.38	**0.002**	-442.91	59.07	-7.50	**< 0.001**

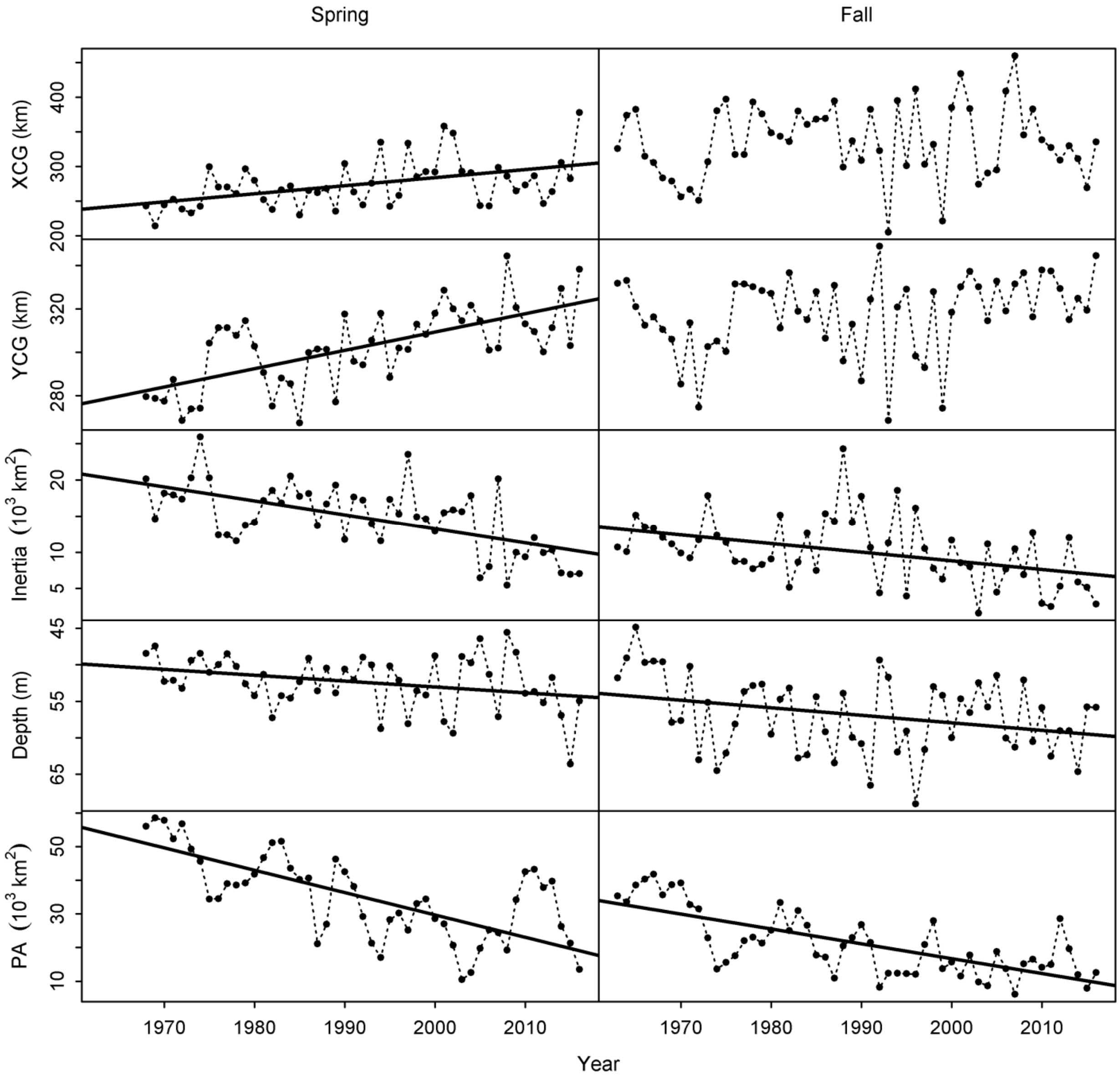

Predictor variables

Time-series of predictor variables for yellowtail flounder (Southern New England-Mid Atlantic). Predictors and associated units are: Northeast Fisheries Science Center (NEFSC) bottom trawl survey stratified mean kg per tow (biomass), catch/NEFSC stratified mean kg per tow (relative *F*) and NEFSC stratified mean bottom temperature (°C). Data for all three predictors were set to NA for years in which there were insufficient bottom temperature recordings to calculate a stratified mean. Sample sizes: spring (n = 46); fall (n = 51).

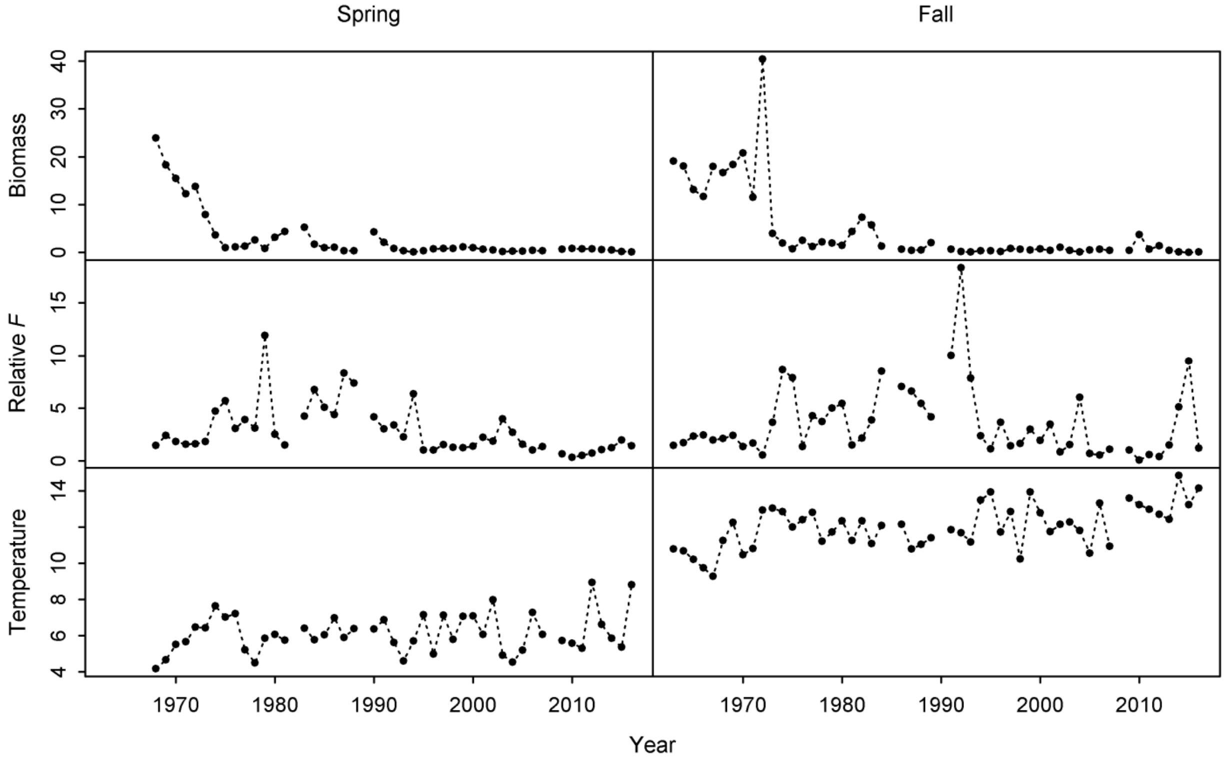

Relative importance of predictors on spatial distribution

Relative importance of predictor variables on spatial indicators for yellowtail flounder (Southern New England-Mid Atlantic). Spatial indicators and associated units are: geographically referenced longitude and latitude of the center of gravity (XCG and YCG, respectively; km), inertia (km^2), depth (m) and positive area (PA; km^2). Predictor variables are: Northeast Fisheries Science Center (NEFSC) bottom trawl survey stratified mean kg per tow (biomass; kg), catch/NEFSC stratified mean kg per tow (relative *F*) and NEFSC stratified mean bottom temperature (°C). Predictor variables with the highest summed Akaike weights for each indicator are in bold.

	**Spring**			**Fall**		
Predictor	Biomass	Relative *F*	Temperature	Biomass	Relative *F*	Temperature
XCG	**0.97**	0.26	0.47	0.26	0.25	**0.29**
YCG	**0.99**	0.28	0.22	0.36	**0.45**	0.24
Inertia	**0.84**	0.69	0.25	0.54	**0.86**	0.48
Depth	**0.61**	0.23	0.39	0.29	0.30	**0.95**
PA	**1.00**	0.22	0.22	**1.00**	0.33	0.91

Model-averaged parameter estimates

Model-averaged predictor estimates by spatial indicator for yellowtail flounder (Southern New England-Mid Atlantic). Spatial indicators and associated units are: geographically referenced longitude and latitude of the center of gravity (XCG and YCG, respectively; km), inertia (km^2), depth (m) and positive area (PA; km^2). Predictor variables are: Northeast Fisheries Science Center (NEFSC) bottom trawl survey stratified mean kg per tow (biomass; kg), catch/NEFSC stratified mean kg per tow (relative *F*) and NEFSC stratified mean bottom temperature (°C).

	**Spring**			**Fall**		
	Biomass	Relative *F*	Temperature	Biomass	Relative *F*	Temperature
XCG	-12.28	1.25	2.89	-0.87	0.91	-1.47
YCG	-7.48	-0.62	-0.04	-0.67	-1.67	-0.13
Inertia	1171.33	1260.67	80.79	401.00	1467.03	-407.92
Depth	-0.47	-0.03	0.24	-0.12	0.18	1.86
PA	7973.86	32.79	2.73	4547.13	274.95	-1434.00
